# Strengthened Interfacial Coupling Between Self‐Assembled Monolayers and Bulk Heterojunctions Enables Thermally Stable Organic Solar Cells

**DOI:** 10.1002/adma.73709

**Published:** 2026-06-14

**Authors:** Gengxin Du, Zhihong Wang, Songyang Yuan, Wenlin Jiang, Shanchao Ouyang, Chengda Ge, Tian Xia, Yiting Jiang, Nan Zhang, Yidan An, Lingyi Ke, Sai Wing Tsang, Francis R. Lin, Qian Li, Alex K.‐Y. Jen, Xuechen Jiao, Yong Zhang, Hin‐Lap Yip

**Affiliations:** ^1^ Department of Materials Science and Engineering City University of Hong Kong Kowloon Hong Kong China; ^2^ School of Materials Science and Engineering Harbin Institute of Technology Harbin China; ^3^ School of Chemistry Guangzhou Key Laboratory of Materials for Energy Conversion and Storage Key Laboratory of Electronic Chemicals for Integrated Circuit Packaging South China Normal University (SCNU) Guangzhou China; ^4^ School of Energy and Environmental Science City University of Hong Kong Kowloon Hong Kong China; ^5^ Hong Kong Institute For Clean Energy (HKICE) City University of Hong Kong Kowloon Hong Kong China; ^6^ Department of Chemistry City University of Hong Kong Kowloon Hong Kong China; ^7^ State Key Laboratory of Marine Environmental Health City University of Hong Kong Kowloon Hong Kong China; ^8^ National Synchrotron Radiation Laboratory the University of Science and Technology of China Hefei China

**Keywords:** hole selective layer, interfacial interactions, organic solar cells, self‐assembled monolayer, thermal stability

## Abstract

Self‐assembled monolayers (SAMs) have emerged as an effective interfacial strategy for improving charge extraction and interfacial energetics in organic solar cells (OSCs); however, limited operational stability, particularly under prolonged high‐temperature conditions, remains a critical challenge for practical deployment. Here, we systematically engineer SAM terminal groups to elucidate how interfacial molecular interactions between the SAM and the bulk‐heterojunction active layer govern device efficiency and thermal stability. Expanding the aromatic ring size of the SAM pendant groups enhances *π–π* and van der Waals interactions, leading to stronger molecular coupling and a more intact and robust interfacial structure at both the electrode/SAM and SAM/active‐layer interfaces. In particular, SAMs incorporating naphthalene pendant groups exhibit significantly strengthened intermolecular interactions, effectively suppressing thermally induced morphological degradation under elevated temperatures. As a result, PM6:BTP‐eC9‐based binary and ternary organic solar cells achieve power conversion efficiencies of 19.73% and 20.15%, respectively. Notably, devices employing this interfacial molecular locking strategy deliver a T_90_ operational lifetime of 150 h under maximum power point tracking at 85°C, representing an order‐of‐magnitude improvement compared to SAMs without pendant groups. These findings establish aromatic terminal group expansion as an effective molecular design strategy for simultaneously enhancing efficiency and thermal stability in organic solar cells.

## Graphical abstract

Molecularly engineered self‐assembled monolayers (SAMs) featuring extended conjugated terminals facilitate superior interfacial coupling with the organic solar cell active layer. By physically anchoring the local morphology, this design suppresses thermal degradation, allowing devices to operate at 85°C for 150 h before degrading to 90% of their initial efficiency—outperforming conventional SAMs by a factor of 15.

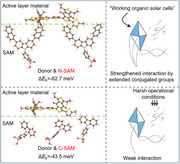

## 1 Introduction

Organic solar cells (OSCs) offer compelling advantages including light weight [1, 2], semi‐transparency [3], solution processability, and compatibility with large‐scale printing technologies [4, 5], which collectively position them as promising candidates for next‐generation renewable energy applications [6]. Recent advances have propelled the highest power conversion efficiency (PCE) of OSCs to approach 21% through the synergistic development of novel materials [7], active layer morphology optimization [8], and sophisticated interface engineering [9], reflecting significant progress toward practical implementation. However, operational stability remains a critical bottleneck for practical application [10, 11], particularly under the elevated temperature conditions encountered during operation [12, 13, 14]. Photovoltaic devices operating under standard solar irradiation can experience surface temperatures reaching over 70°C [15], which imposes severe constraints on the thermal robustness of both the active layer and interfacial components. While previous studies have addressed OSC stability under ultraviolet exposure [16], thermal aging [17], and general operating conditions [18], leading to extended device lifetimes and improved understanding of degradation mechanisms, the operational stability of OSCs under high‐temperature environments (≥85°C) with concurrent solar irradiation and thermal stress remains largely unexplored.

The conventional OSC architecture comprises anode, hole‐selective layer (HSL), active layer, electron‐selective layer (ESL), and cathode, where the HSL and ESL serve as critical interfacial layers that regulate energy‐level alignment and facilitate efficient charge extraction between the electrodes and the active layer. Over the past years, self‐assembled monolayers (SAMs) have emerged as superior HSL materials, with numerous high‐efficiency OSCs incorporating SAM‐based interfaces. Compared with conventional hole transport materials such as PEDOT:PSS, SAMs offer precise energy‐level tunability, reduced hygroscopicity, mitigated electrode corrosion, and suppressed parasitic currents [7, 9, 19, 20, 21, 22]. SAM molecules feature a tripartite architecture consisting of an anchoring head group, a molecular linker, and a functional terminal group [23]. The anchoring moiety (typically phosphonic acid groups) forms robust covalent bonds with the ITO substrate, while the linker segment connects the anchoring and terminal groups—with rigid aromatic linkers generally providing improved molecular ordering and device stability [16]. The functional terminal group offers the greatest structural versatility, directly influencing molecular energy levels, conformational packing, and interfacial interactions with the overlying active layer [24, 25]. This modular molecular design offers substantial opportunities to simultaneously optimize device efficiency and operational stability in SAM‐based OSCs.

Thermal degradation of OSCs primarily stems from morphological evolution within the active layer and functional failure at critical interfaces [26]. Previous approaches to mitigate high‐temperature degradation have focused on developing thermally stable active layer materials [18] or implementing robust interfacial layers such as SAM‐based HSLs [12, 27]. Recent molecular engineering efforts have systematically optimized SAM structures to enhance interfacial stability under elevated temperatures and diverse operating conditions [16, 24, 28], thereby extending device operational lifetimes [13, 29]. Importantly, studies have demonstrated that SAMs can establish strong interactions with the active layer, leading to substantially prolonged device stability under continuous solar illumination compared to conventional interfaces [25, 30]. These findings suggest that strengthened interfacial interactions can effectively constrain active‐layer phase evolution under thermal stress. However, the fundamental relationship between molecular‐level interfacial interactions and macroscopic device stability has not yet been fully elucidated.

Herein, we present a systematic molecular design strategy comprising a series of SAMs in which the carbazole terminal group is functionalized with aromatic pendant units—phenyl (P) and naphthyl (N)—yielding C‐SAM (control without pendant), P‐SAM, and N‐SAM molecules, all connected to a phosphonic acid head group through a rigid phenyl backbone. This stepwise aromatic expansion enables controlled tuning of interfacial coupling while preserving identical anchoring chemistry and backbone rigidity, thereby establishing a well‐defined platform to examine how increasing aromaticity at the SAM/active‐layer interface governs molecular packing, interfacial interactions, and morphological stability under thermal stress. Within this framework, systematic interfacial characterization reveals a clear dependence of interfacial organization on terminal‐group aromaticity. Notably, N‐SAM, featuring a naphthyl‐substituted carbazole terminal group, exhibits the highest packing density, favorable energy‐level alignment, and the strongest interfacial interactions with the bulk‐heterojunction layer, as supported by adhesion energy analysis, surface potential mapping, and phase imaging. Consequently, N‐SAM‐based devices achieve high efficiencies while maintaining significantly improved operation at elevated temperatures (up to 85°C), underscoring the role of aromatic pendant group engineering in enabling thermally robust organic solar cells.

## 2 Results and Discussion

**FIGURE 1:**
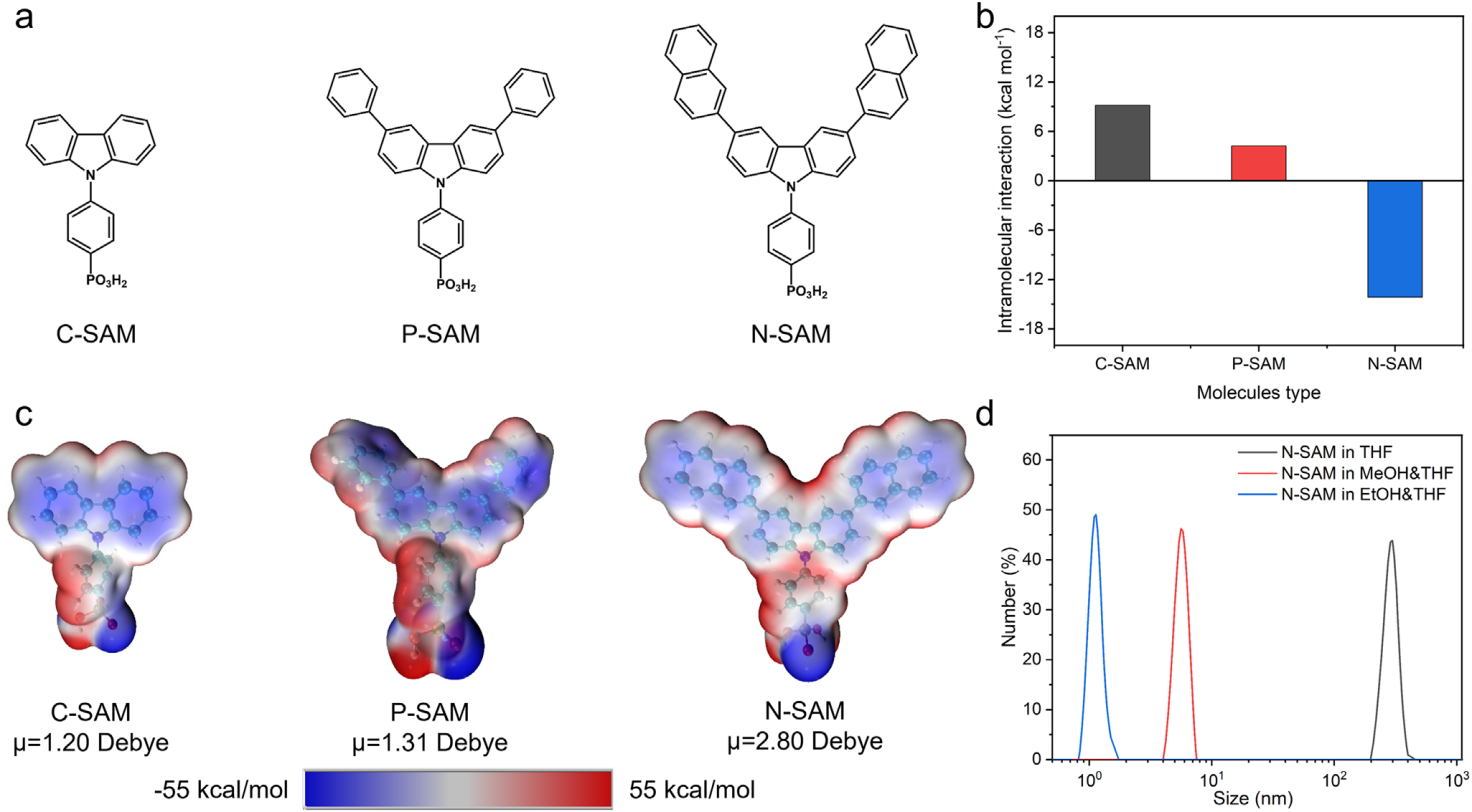
Molecular design and physicochemical characterization of SAMs. (a) Chemical structures of C‐SAM, P‐SAM, and N‐SAM, illustrating the systematic extension of the conjugated terminal group. (b) DFT‐calculated intermolecular interaction energies for the three SAM variants. (c) ESP maps and corresponding calculated molecular dipole moments of the SAM molecules. (d) DLS analysis of N‐SAM dispersion in pure THF, methanol/THF azeotrope, and ethanol/THF azeotrope.

2PACz is a benchmark SAM widely employed in OSCs to enhance charge extraction efficiency and has been recognized as an effective strategy for improving PCE [7, 9, 22]. However, 2PACz suffers from limited stability under photovoltaic operating conditions [11], primarily due to intrinsic photochemical instability and undesirable molecular aggregation behavior [31, 32]. To overcome these limitations, we systematically designed a series of SAM molecules with enhanced structural rigidity, stability, and interfacial performance. Specifically, the flexible alkyl linker in 2PACz was first replaced with a rigid aromatic backbone to improve photostability, yielding a carbazole‐based SAM denoted as C‐SAM. Building on this framework, the conjugated architecture was further expanded through the symmetric incorporation of additional aromatic units at the meta positions of the carbazole group, producing P‐SAM (featuring phenyl pendant groups) and N‐SAM (featuring naphthyl pendant groups). The molecular structures of this systematic series are presented in Figure 1a.

Progressive extension of the conjugated ring system strengthens intermolecular π‐π stacking interactions, which are expected to influence both molecular packing and interfacial organization. Density functional theory (DFT) calculations were performed to study the optimized molecular conformations in free space (Figure S1), enabling quantitative evaluation of intermolecular interaction energies (Figure 1b), where negative values indicate attractive interactions and positive values denote repulsion. Among the three SAMs, N‐SAM exhibits the strongest aggregation tendency, with a pronounced attractive interaction energy of −14.16 kcal mol^−^^1^, whereas P‐SAM and C‐SAM show progressively weaker interactions, characterized by repulsive energies of 4.22 and 9.15 kcal mol^−^^1^, respectively.

To further elucidate the structure–property relationship, the electrostatic potential (ESP) maps for C‐SAM, P‐SAM, and N‐SAM were analyzed (Figure 1c). Expansion of the π‐conjugated terminal group from unsubstituted carbazole in C‐SAM to phenyl‐substituted carbazole in P‐SAM and further to the larger naphthyl‐substituted carbazole in N‐SAM leads to increased charge delocalization across the molecule, resulting in a more homogeneous ESP despite the concomitant increase in molecular dipole moment from 1.20 to 1.31 D and 2.80 D. This delocalized and smoothly varying surface potential reduces localized charge polarization and promotes a well‐oriented interfacial dipole, thereby facilitating stronger and more directional coordination between the phosphonic acid anchoring group and surface metal–oxygen sites on ITO. Consistent with XPS‐derived surface binding and adhesion energy analyses, as discussed later, N‐SAM forms a more stable and robust covalent interface with ITO, leading to improved interfacial integrity and more effective surface modification.

While extended π–conjugation enhances interfacial interactions, it simultaneously introduces challenges associated with solubility and aggregation. Initial solution processing using isopropanol/toluene azeotropes, optimized in our previous work [30], resulted in poor solubility and pronounced aggregation of N‐SAM, leading to visibly turbid solutions (Figure S2). To address this issue, THF‐based azeotropic solvent systems were systematically investigated because THF provides a balanced solvation environment: its moderate polarity and Lewis basicity enable effective interaction with the polar phosphonic acid anchoring group, while its cyclic ether structure affords sufficient dispersive interactions to solvate the extended π‐conjugated aromatic framework. This dual solvation capability suppresses strong intermolecular association and promotes improved molecular dispersion. Although C‐SAM and P‐SAM exhibited good solubility in both ethanol/THF and methanol/THF azeotropes, the stronger *π–π* interactions in N‐SAM necessitated a carefully optimized ethanol/THF composition to achieve uniform dispersion.

Dynamic light scattering (DLS) measurements provide direct evidence of this solvent‐dependent dispersion behavior. As shown in Figure 1d, N‐SAM forms large in large aggregates in pure THF (∼300 nm), evolves into micelle‐like assemblies in methanol/THF (∼6 nm), and ultimately reaches near‐molecular dispersion in ethanol/THF (∼1 nm). The importance of solvent engineering is further validated at the device level (Table S1), where progressive improvement in N‐SAM dispersion leads to a corresponding increase in photovoltaic performance, with PCE rising from 13.86% (pure THF) to 19.11% (methanol/THF) and further to 19.73% (ethanol/THF). Collectively, these results demonstrate that precise control of molecular aggregation through solvent optimization is critical for realizing the full interfacial and device‐level advantages of highly conjugated SAMs.

**FIGURE 2:**
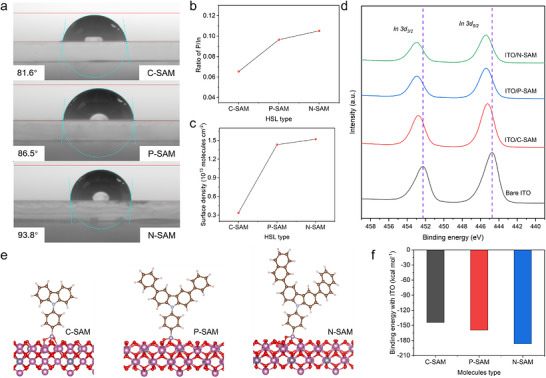
Surface characterization and theoretical analysis of SAM‐modified ITO substrates. (a) Static water contact‐angle measurements for ITO surfaces modified by C‐SAM, P‐SAM, and N‐SAM. (b) Phosphorus‐to‐indium (P/In) elemental ratios determined by XPS for different SAM‐modified ITOs. (c) Surface molecular densities extracted from CV measurements.(d) High‐resolution XPS spectra of the indium 3d core level for ITO surfaces modified by different SAMs.(e) DFT‐optimized molecular conformations of C‐SAM, P‐SAM, and N‐SAM on ITO surfaces.(f) Calculated binding energies between SAM molecules and the ITO substrate.

To investigate the fundamental properties of these SAMs, slot‐die coating was employed to deposit uniform SAM layers from well‐dispersed ethanol/THF azeotropic solutions. Contact‐angle measurements using deionized water droplets were conducted to evaluate surface wettability and film quality (Figure 2a). The contact angle increases systematically from C‐SAM (81.6°) to P‐SAM (86.5°) and further to N‐SAM (93.8°), which can be attributed to two complementary factors: (i) the progressively increased aromatic content of the terminal headgroups, which enhances nonpolar surface character as the conjugated framework extends from pure carbazole to naphthalene‐substituted carbazole, and (ii) increasingly dense molecular packing on the ITO surface.

To quantitatively evaluate SAM surface coverage and molecular packing density, x‐ray photoelectron spectroscopy (XPS) and electrochemical surface density measurements based on cyclic voltammetry (CV) were employed. XPS analysis provided the phosphorus‐to‐indium (P/In) elemental ratio (Figure 2b, Figure S3, and Table S2), where phosphorus originates from the SAM anchoring group and indium serves as a characteristic marker of the ITO substrate. The P/In ratio of 10.5% for N‐SAM is significantly higher than that of P‐SAM (9.7%) and C‐SAM (6.5%), indicating the highest packing density and surface coverage for the N‐SAM. Electrochemical surface density measurements further corroborate this trend, revealing consistent and progressively increased molecular coverage patterns across the three SAM systems (Figure 2c, Figure S4). The enhanced packing density of N‐SAM can be attributed to their stronger intermolecular π‐π interactions and increased chemical affinity toward the ITO surface, enabled by the extended π‐conjugated system, which promotes more ordered self‐assembly when deposited from the optimized azeotropic solvent system.

High‐resolution XPS analysis of the indium 3d core levels was conducted to quantitatively assess the anchoring strength of the SAMs on ITO substrates (Figure 2d). Compared to bare ITO, systematic binding‐energy shifts in both the In 3d_3/2_ and In 3d_5/2_ peaks are observed after SAM modification, confirming successful anchoring through covalent bond formation. Notably, ITO/N‐SAM exhibits the highest binding energies, with values of 452.97 eV (In 3d_3/2_) and 445.43 eV (In 3d_5/2_), compared to ITO/P‐SAM (452.94 and 445.40 eV), and ITO/C‐SAM (452.79 and 445.25 eV). This progressive increase in indium binding energies correlates directly with the degree of π‐conjugation in the SAM terminal groups, indicating that N‐SAM forms the strongest interfacial bonding with ITO. The enhanced anchoring strength is attributed to the increased acidity of the phosphonic acid anchoring groups induced by the extended π‐conjugated system, which strengthens initial acid‐base interactions with surface metal‐oxygen sites on the ITO surface and subsequently promotes condensation reactions to form robust covalent P─O─M bonds, leading to more densely packed molecular assemblies [33].

To gain deeper mechanistic insights into the anchoring behavior, density functional theory (DFT) calculations were performed to model the molecular orientations of the three SAMs on ITO and to compute their theoretical binding energies (Δ*E*_b_). The optimized molecular conformations reveal that all three SAMs adopt a tridentate bonding mode on the ITO surface (Figure 2e), while their inherent molecular rigidity prevents conformational collapse and stabilizes an upright adsorption geometry. Notably, the large dipole moment of N‐SAM promotes a more perpendicular molecular orientation, which maximizes exposure of the π‐conjugated terminal groups toward the overlying active layer. Consistent with experimental XPS observations, N‐SAM exhibits the most favorable calculated binding energy (Δ*E*_b_ = ‐186.6 kcal mol^−1^) (Figure 2f), confirming its superior thermodynamic stability and strongest substrate adsorption among the three SAM variants.

**FIGURE 3:**
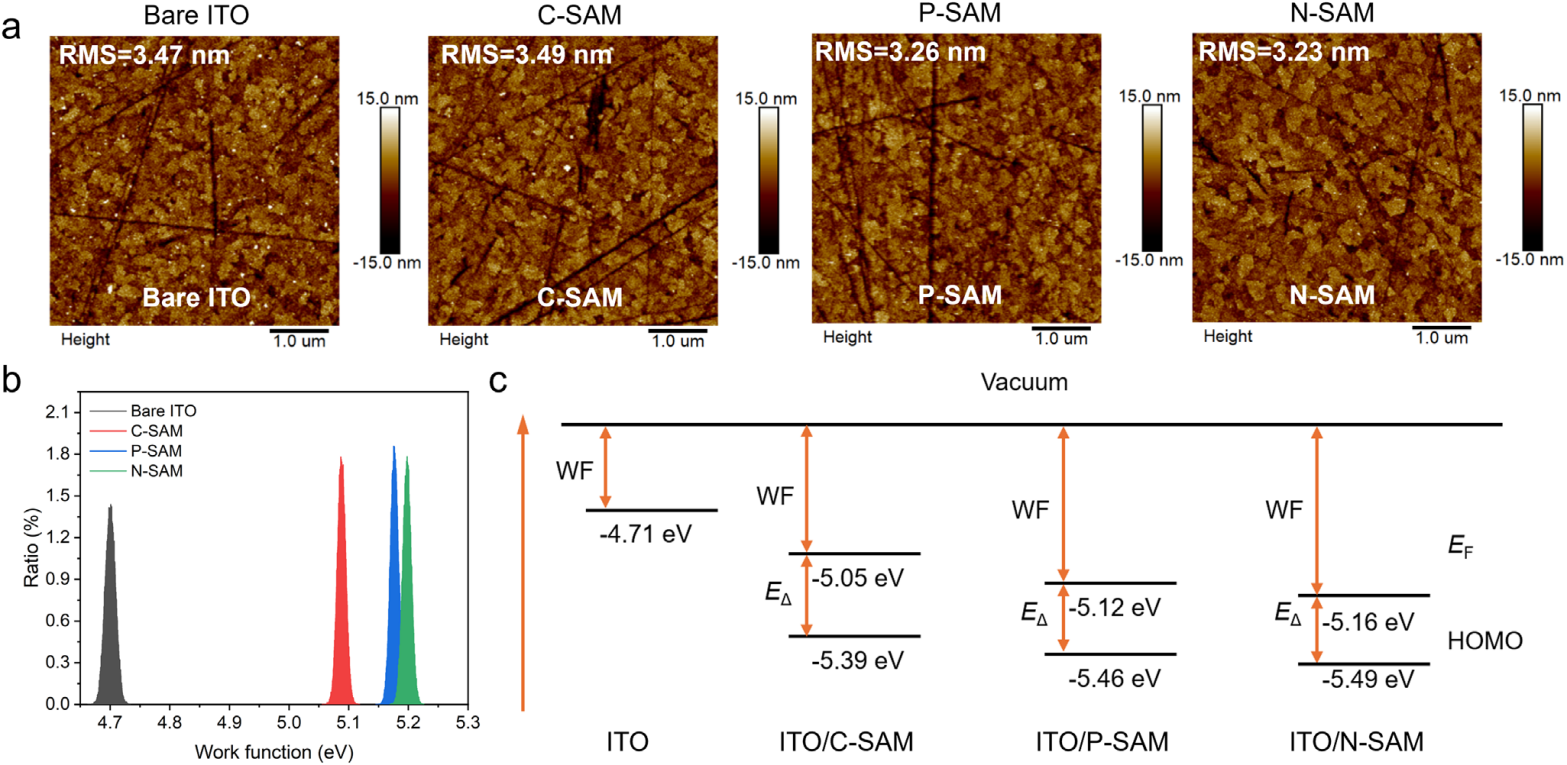
Surface morphology and energetic characterization of SAM‐modified ITO electrodes.(a) AFM height images of bare ITO and SAM‐modified ITO substrates.(b) KPFM surface potential distributions of bare ITO and SAM‐modified ITO substrates.(c) Energy level diagrams for bare ITO and SAM‐modified ITO electrodes. (WF, E_F_, and E_∆_ represent the work function, Fermi level, and energy offset between E_F_ and HOMO, respectively).

Having established the superior molecular conformation and deposition quality of the SAM films, we employed a comprehensive suite of surface characterization techniques, including atomic force microscopy (AFM), Kelvin probe force microscopy (KPFM), and ultraviolet photoelectron spectroscopy (UPS), to evaluate their suitability as HSL in OSCs (the transmission spectra for ITO/SAMs structure was measured and shown in Figure S5). AFM topographical analysis revealed that all three SAMs form conformal coatings on the ITO substrate while maintaining surface roughness comparable to bare ITO (root mean square (RMS) roughness = 3.47 nm) (Figure 3a). A marginal reduction in surface roughness is observed for P‐SAM (RMS = 3.26 nm) and N‐SAM (RMS = 3.24 nm), which is attributed to their enhanced surface coverage and denser molecular packing, consistent with the XPS and CV results discussed above.

KPFM measurements were conducted to probe the surface contact potential distributions of the SAM‐modified ITO substrates, using bare ITO as a reference (Figure S6). The resulting potential maps exhibit excellent spatial uniformity across all SAM‐modified surfaces (Figure 3b), confirming homogeneous film formation. Notably, the work function increased systematically with increasing SAM conjugation, from −5.09 eV for C‐SAM to −5.17 eV for P‐SAM and −5.20 eV for N‐SAM‐modified ITO, indicating that greater π‐conjugation enhances the work function modulation capability. These trends are independently confirmed by UPS measurements (Figure 3c and Figure S6), which reveal consistent work function shifts.

Crucially, the N‐SAM‐modified ITO exhibits the most favorable interfacial energy‐level alignment, featuring a work function of −5.16 eV determined from UPS measurements and a highest occupied molecular orbital (HOMO) level of −5.49 eV derived from UPS analysis (Figure S7), which closely matches the ionization potential of the PM6 donor polymer (the energy level of lowest unoccupied molecular orbital (LUMO) was estimated from the optical absorption edge; see Figure S8). This optimized energy level alignment facilitates efficient hole extraction and is expected to maximize the open‐circuit voltage (*V*_OC_) in OSC devices. Collectively, these surface characterization results demonstrate that N‐SAM possesses superior interfacial electronic properties for HSL applications, outperforming both P‐SAM and C‐SAM counterparts.

More importantly, the influence of SAM molecular design on interlayer interactions and thermal robustness was systematically investigated. To quantitatively evaluate the interfacial adhesion between SAMs and the active layer, a peel‐off test methodology was employed, in which PM6 donor polymer films were spin‐coated onto SAM‐modified ITO substrates and subsequently detached using a tensile testing apparatus. PM6 serves as the hole‐transporting donor component in the active layer, forming the primary charge‐extraction pathway between the bulk heterojunction and the SAM‐modified ITO electrode.

**FIGURE 4:**
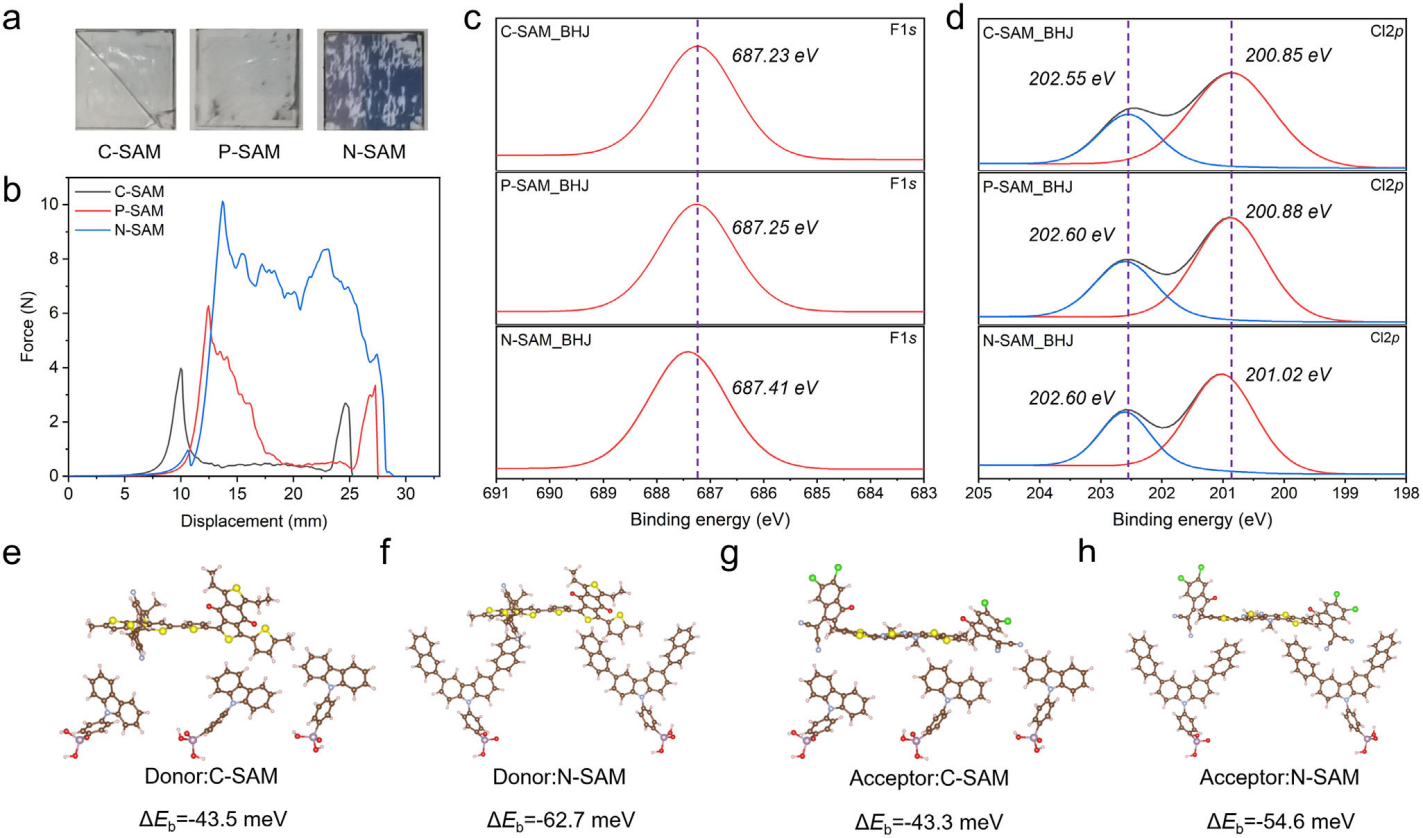
Interfacial interaction analysis. (a) Optical images of ITO/SAM substrates after peel‐off removal of the blue‐colored PM6 films. (b) Force‐displacement curves obtained from mechanical peel‐off tests of PM6 films on different SAM‐modified ITO substrates. High‐resolution XPS spectra of the F1s (c) and Cl2p (d) core levels for SAM‐deposited PM6:BTP‐eC9 BHJ films. DFT‐calculated binding configurations and energies between a simplified PM6 donor segment and C‐SAM (e) and N‐SAM (f). DFT‐calculated binding configurations and energies between a simplified BTP‐eC9 acceptor molecule and C‐SAM (g) and N‐SAM (h).

Visual inspection of the substrates following the peel‐off testing reveals distinct adhesion behaviors, readily evidenced by the residual blue PM6 donor polymer film on the ITO surface (Figure 4a). Specifically, the blue PM6 layer was completely removed from C‐SAM‐modified ITO, nearly completely detached from P‐SAM‐modified ITO, and only partially removed from N‐SAM‐modified ITO, indicating markedly stronger interfacial adhesion in the latter case. Quantitative analysis was performed using force‐displacement measurements acquired during the peel‐off tests on 15 mm × 15 mm ITO substrates (Figure 4b). The corresponding adhesion energies, calculated by integrating the force‐displacement curves, were determined to be 47, 120, and 473 J/m^2^ for C‐SAM, P‐SAM, and N‐SAM, respectively. This nearly ten‐fold increase in adhesion energy from C‐SAM to N‐SAM unambiguously demonstrates that incorporating extended π‐conjugated moieties into the SAM terminal groups substantially strengthens interfacial π–π stacking interactions with the conjugated donor polymer backbone.

To gain insight into the interfacial interactions between the SAMs and the bulk heterojunction (BHJ) active layer composed of PM6 donor and BTP‐eC9 acceptor, a reverse‐deposition architecture was employed as an indirect probe of interfacial chemistry. In this configuration, SAM solutions were deposited onto pre‐formed active‐layer films, allowing changes in the local chemical environment of the active‐layer components induced by SAM contact to be detected [34]. High‐resolution XPS analysis of the F 1s peak, characteristic of the PM6 donor, reveals a systematic shift toward higher binding energy with increasing SAM conjugation, from 687.23 eV for C‐SAM to 687.41 eV for N‐SAM (Figure 4c). Although this structure does not replicate the full device architecture, the observed binding‐energy shifts provide indirect yet compelling evidence of strengthened interfacial electronic interactions. The shift toward higher binding energy is attributed to enhanced *π–π* coupling between the conjugated SAM terminal groups and the PM6 backbone, which reduces the local electron density at the fluorinated moieties. The larger shift observed for N‐SAM therefore reflects stronger electronic coupling, consistent with its extended π‐conjugated structure and superior interfacial affinity.

Complementary analysis of the Cl 2p peak, characteristic of the BTP‐eC9 acceptor, exhibits a similar binding energy shift (Figure 4d), confirming that SAMs with extended π‐conjugated terminal groups enhance interfacial interactions with both donor and acceptor components. This behavior arises from the compatible π‐conjugated architectures of the SAM terminal groups and the active‐layer materials (Figure S9). These experimental observations are supported by DFT calculations, in which alkyl side chains were truncated to reduce computational complexity while preserving the essential π‐conjugated cores. The calculated binding energies (Δ*E*_b_) between SAMs and active‐layer components increase systematically from C‐SAM to N‐SAM, from −43.5 to −62.7 meV for SAM–donor interactions (Figure 4e,f) and from −43.3 to −54.6 meV for SAM–acceptor interactions (Figure 4g,h). Notably, N‐SAM exhibits a preferential binding affinity toward the donor polymer, with a significantly larger donor–acceptor binding‐energy contrast than observed for C‐SAM and P‐SAM (Figure S10). This selective binding preference is advantageous for promoting donor‐rich interfacial regions near the ITO electrode, thereby optimizing the vertical phase distribution for efficient hole extraction.

To examine how enhanced interfacial interactions affect morphological stability under thermal stress, half‐cell devices were prepared by depositing BHJ active layers onto bare ITO and various SAM‐modified ITO substrates. Selected samples were subjected to thermal aging at 85°C for 96 h on a temperature‐controlled hot plate, while the remaining samples served as fresh controls. Both fresh and aged samples were then analyzed using XPS to determine the elemental composition at the buried SAM/BHJ interface and at the top surface, thereby clarifying the role of different SAMs in BHJ morphological evolution and thermal stability.

**FIGURE 5:**
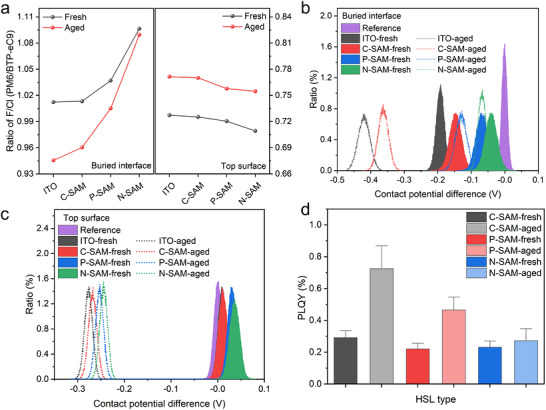
Thermal stability analysis of SAM‐modified OSC interfaces. XPS‐determined F/Cl elemental ratios at the buried interface (a, left) and top surface (a, right) of BHJ films on bare and SAM‐modified ITO substrates, comparing fresh and thermally aged samples (85°C, 96 h). KPFM contact potential distributions measured at the buried interface (b) and top surface (c) before and after thermal aging. (d) PLQY of fresh and thermally aged ITO/SAM/BHJ structures.

Elemental analysis of the buried interface reveals systematic variations in the fluorine‐to‐chlorine (F/Cl) ratio (Figure 5a), with fluorine and chlorine serving as characteristic markers for the PM6 donor and BTP‐eC9 acceptor, respectively. In fresh samples, the F/Cl ratio increases progressively from bare ITO (1.012) to N‐SAM‐modified substrates (1.095), indicating that the enhanced π–π interactions provided by N‐SAM preferentially enrich PM6 molecules at the ITO interface compared with other SAM variants or bare ITO (Figure S11). This donor‐enriched interfacial region near the anode is favorable for suppressing interfacial recombination and promoting efficient hole transport, thereby contributing to improved device performance.

After thermal aging, all samples exhibit a decrease in the F/Cl ratio at the buried interface, indicating thermally induced migration of BTP‐eC9 acceptor molecules toward the anode. Such acceptor migration reflects phase degradation and morphological instability within the heterojunction near the buried electrode interface, which are well‐known contributors to long‐term device instability in OSCs. Notably, samples incorporating extended π‐conjugated SAM terminal groups show markedly smaller compositional changes, with N‐SAM displaying minimal variation (from 1.095 to 1.089). This improved thermal stability is attributed to simultaneously strengthened interfacial interactions with both donor and acceptor components, which generate a phase‐anchoring effect that mitigates thermally driven phase separation and morphological rearrangements, even under prolonged aging at 85°C.

At the top surface (Figure 5a), the F/Cl ratios for samples ranging from bare ITO to N‐SAM‐modified substrates are very similar (≈0.73–0.71), indicating that the presence of SAMs does not significantly alter the initial surface composition in the absence of direct interfacial contact. After thermal aging, the absolute F/Cl values for all samples increase by approximately 0.04, corresponding to a higher donor‐to‐acceptor ratio at the top surface. This uniform increase suggests a slight redistribution of BTP‐eC9 acceptor molecules away from the film surface and toward the film interior (bulk) during thermal stress, rather than a SAM‐dependent effect. Together with the buried‐interface results, these observations support a degradation scenario in which acceptor molecules undergo thermally driven redistribution, while SAM‐induced interfacial interactions primarily regulate composition and stability at the buried electrode interface.

To quantitatively assess the energetic evolution of the BHJ active layer under thermal stress, KPFM measurements were performed on BHJ films before and after thermal aging at 85°C (Figure 5b,c). Contact potential difference measurements were collected at both the buried interface and the top surface, using a gold film as the reference electrode.

At the buried interface, samples with strong interfacial interactions exhibit markedly enhanced energetic stability upon high‐temperature aging. In particular, N‐SAM‐modified devices show minimal contact‐potential shifts (∼0.03 V), while P‐SAM‐modified devices display slightly larger but still modest changes (∼0.06 V) (Figure 5b). In contrast, devices with weak interfacial *π–π* interactions, including bare ITO and C‐SAM‐modified substrates, undergo substantial contact‐potential variations exceeding 0.3 V, indicative of severe interfacial energetic perturbations. Such pronounced energetic instability is expected to increase energy barriers for charge generation and transport, thereby adversely affecting device performance.

By comparison, analysis of the top surface region reveals that the nature of the bottom hole‐selective SAM layer has little influence on the energetic evolution of the film surface. All samples experience similar potential shifts after thermal aging, independent of the underlying SAM interactions (Figure 5c). This observation confirms that the stabilizing “phase‐anchoring” effect imparted by strongly interacting SAMs is spatially localized at the buried interface. Consistent with this conclusion, AFM phase imaging reveals minimal thermally induced phase separation at the buried interface for systems incorporating SAMs with extended terminal groups, providing further evidence of their superior morphological stabilization (Figure S12).

To further elucidate the effects of thermal aging on the nanoscale morphology and phase behavior of the BHJ layer, photoluminescence quantum yield (PLQY), transient absorption (TA) spectroscopy, and resonant soft x‐ray scattering (RSoXS) were employed as complementary probes of thermally induced phase evolution. Photoluminescence originates from radiative recombination of excitons that fail to undergo charge separation due to insufficient donor‐acceptor interfacial area and therefore remains low in well‐mixed, freshly prepared BHJ films. Upon prolonged thermal aging at 85°C for 96 h, the increased thermal energy promotes morphological coarsening and phase segregation, leading to a systematic increase in PLQY across all aged samples (Figure 5e, Figures S13 and S14). This PLQY increase is indicative of enlarged domains that reduce the effective interfacial area available for exciton dissociation.

Notably, devices incorporating N‐SAM as the HSL exhibit substantially enhanced morphological stability, with PLQY increasing only slightly from 0.24% to 0.28% after aging. In contrast, significantly larger PLQY increases are observed for P‐SAM (>0.2% increase) and C‐SAM (>0.4% increase) systems. These results highlight the critical role of strong interfacial interactions enabled by the extended π‐conjugated architecture of N‐SAM in suppressing thermally driven phase segregation [34].

Transient absorption (TA) spectroscopy was employed to investigate the charge carrier dynamics in the PM6:BTP‐eC9 blend films deposited on different SAM‐modified substrates (Figure S15). The rise dynamics of ground‐state bleaching signals corresponding to PM6 (GSB_D_) exhibit the order of hole transfer efficiency as N‐SAM>P‐SAM>C‐SAM, indicating that N‐SAM facilitates faster and more efficient hole transfer from BTP‐eC9 to PM6, thereby mitigating geminate recombination. Notably, even after aging, the same trend is preserved among the three SAMs, suggesting that the SAM‐induced interfacial properties remain effective in promoting charge extraction under thermal aging.

RSoXS measurements provide further insight into the morphological evolution of the BHJ films. All samples exhibit hierarchical phase separation, as evidenced by the coexistence of low‐q and high‐q scattering peaks (Figure S16a) [35, 36], consistent with multiscale morphological organization. Quantitative analysis of domain sizes, extracted from the scattering peak positions, reveals that N‐SAM‐modified devices maintain smaller domains and experience minimal thermal coarsening (from 24.6 to 25.2 nm) after aging. By comparison, C‐SAM‐based counterparts (from 24.7 nm to 27.1 nm) (Figure S16b, Table S3). By maintaining smaller domain sizes, N‐SAM systems enable excitons to reach donor–acceptor interfaces more efficiently, thereby reducing geminate recombination losses [37].

Beyond domain size, RSoXS analysis of the integrated scattering intensity reveals that N‐SAM‐modified devices maintain higher domain purity throughout the thermal aging process compared to C‐SAM systems (Figure S16c, Table S3) [38]. Collectively, the PLQY and RSoXS results demonstrate that incorporating extended π‐conjugated moieties into SAM terminal groups effectively mitigates thermally induced phase degradation in BHJ active layers, thereby preserving the nanoscale morphology essential for stable, high‐temperature operation.

**FIGURE 6:**
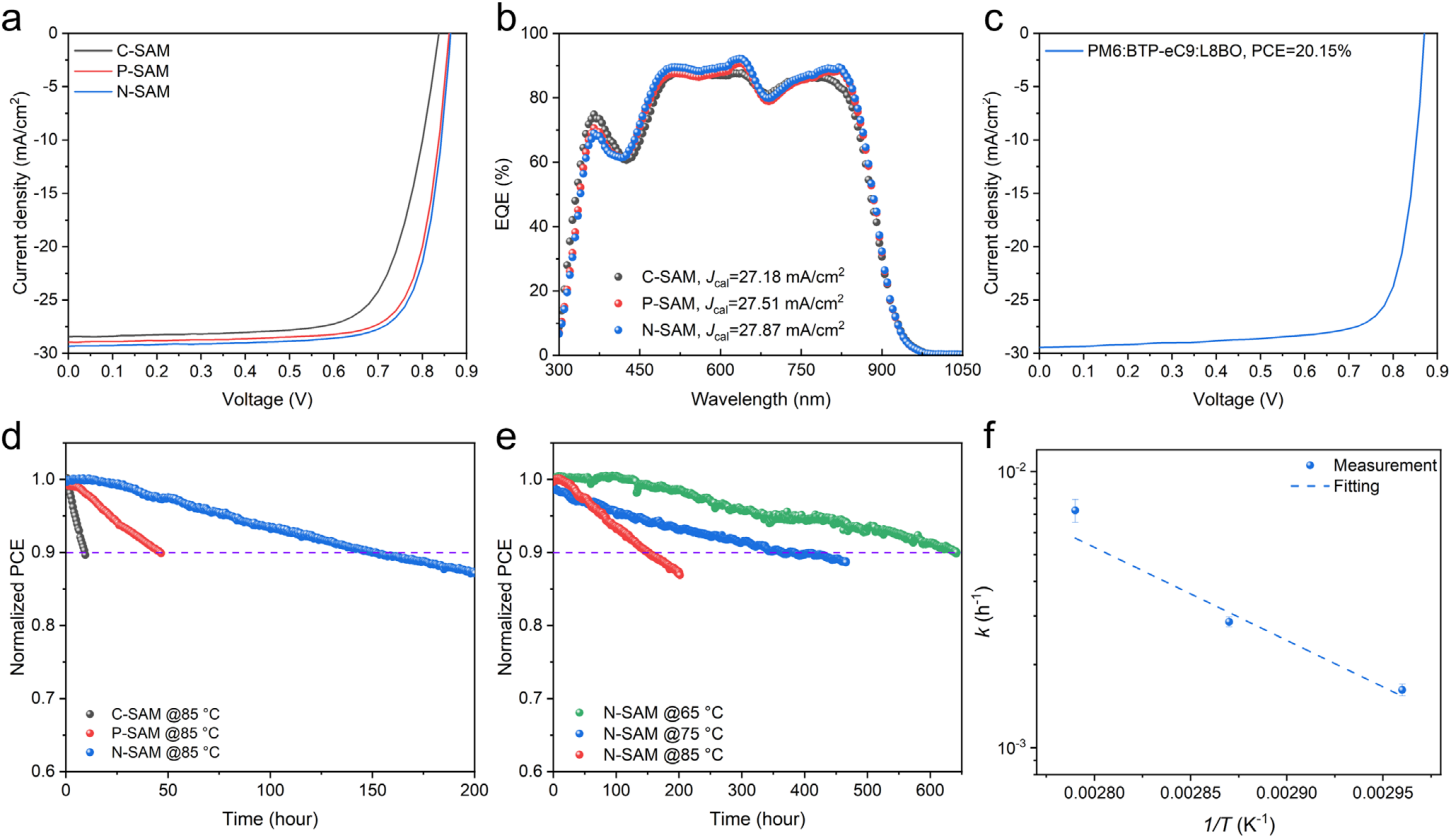
Photovoltaic performance and thermal stability of SAM‐based OSCs. *J–V* curves (a) and EQE spectra (b) for PM6:BTP‐eC9‐based devices with different SAM HSLs. (c) *J–V* curves for PM6:BTP‐eC9:L8BO ternary devices with N‐SAM HSL. (d) MPPT stability at 85°C for devices with different SAM HSLs. (e) Temperature‐dependent MPPT stability for N‐SAM‐based devices. (f) Arrhenius analysis of the degradation kinetics, showing the degradation rate (1/T_90_) as a function of inverse temperature.

To validate the impact of enhanced interfacial interactions on photovoltaic performance, we fabricated OSCs with the architecture ITO/SAM/PM6:BTP‐eC9/PNDIT‐F3N/Ag. Key photovoltaic parameters are summarized in Table 1, and representative current density‐voltage (*J*–*V*) characteristics are shown in Figure 6a. Devices incorporating N‐SAM exhibit the highest open‐circuit voltage (*V*_OC_) and enhanced short‐circuit current density (*J*_SC_), consistent with improved energy‐level alignment and more efficient hole extraction enabled by favorable HOMO matching (Figure S17) [39, 40].

**TABLE 1:** Photovoltaic parameters of OPV devices under the illumination of AM 1.5G, 100 mW cm^−2^.

SAM	BHJ	*V*_OC_ (V)	FF (%)	*J*_SC _(mA/cm^2^)	PCE (%)
C‐SAM	PM6:BTP‐eC9	0.848 (0.847 ± 0.003)	75.85 (73.61 ± 1.81)	28.61 (28.71 ± 0.39)	18.40^a^ (17.90 ± 0.28)^b^
P‐SAM	PM6:BTP‐eC9	0.861 (0.859 ± 0.002)	77.40 (76.91 ± 0.50)	28.94 (28.91 ± 0.15)	19.28 (19.09 ± 0.13)
N‐SAM	PM6:BTP‐eC9	0.864 (0.862 ± 0.001)	77.88 (77.47 ± 0.38)	29.33 (29.15 ± 0.14)	19.73 (19.48 ± 0.19)
N‐SAM	PM6:BTP‐eC9:L8BO	0.870 (0.869 ± 0.003)	78.61 (78.45 ± 0.57)	29.45 (29.32 ± 0.06)	20.15 (19.99 ± 0.11)

^a^ The maximal values.

^b^ average values with standard deviation derived from 10 independent devices.

In addition, the higher molecular packing density associated with P‐SAM and N‐SAM leads to improved interfacial quality, as reflected by substantially higher fill factor (FF) values compared to C‐SAM‐based devices. As a result of the combined enhancements in *V_OC_*, *J_SC_*, and FF, N‐SAM‐based binary OSCs deliver the highest PCE of 19.73%. External quantum efficiency (EQE) measurements further confirm improved photocurrent generation across the main absorption range (450–820 nm), with N‐SAM devices achieving the highest integrated current density of 27.87 mA cm^−^^2^, compared with 27.51 mA cm^−^^2^ for P‐SAM and 27.18 mA cm^−^^2^ for C‐SAM devices (Figure 6b). This enhancement is consistent with stronger *π–π* interactions between N‐SAM and the donor polymer, which promote donor enrichment near the anode and facilitate efficient charge extraction.

Dark current measurements reveal that the denser molecular assemblies formed by P‐SAM and N‐SAM effectively suppress leakage currents and reduce potential shunt pathways (Figure S18). The exciton dissociation efficiency (*η*_diss_) was determined from the relationship between photocurrent (*J*_ph_) and effective voltage (*V*_eff_) (Figure S19), and the devices using N‐SAM as the HSL exhibited high exciton dissociation efficiencies both before and after aging (*η*_diss_ = 0.913 and 0.879, respectively), with the smallest variation among the three SAM‐based devices (0.034 for N‐SAM, 0.056 for P‐SAM, and 0.065 for C‐SAM) after aging, which further supports the findings revealed by PLQY and RSoXS. To further elucidate charge‐recombination dynamics, light‐intensity‐dependent measurements were performed. The nearly identical slopes (∼0.999) observed in *J_SC_* vs. light intensity for all SAM variants indicates negligible bimolecular recombination losses (Figure S20) [41]. Analysis of *V*_OC_ as a function of light intensity further shows that devices based on N‐SAM (1.22 kT/q) and P‐SAM (1.34 kT/q) exhibit significantly reduced trap‐assisted recombination compared to C‐SAM‐based devices (1.51 kT/q) (Figure S21) [42]. These results demonstrate that enhanced SAM–BHJ interfacial interactions effectively passivate interfacial defects and optimize charge‐carrier dynamics.

Finally, to demonstrate the generality of this interfacial engineering strategy, N‐SAM was applied to a range of high‐performance active‐layer systems, including D18:BTP‐eC9 and PM6:L8BO blends (Figure S22 and Table S4). In both systems, N‐SAM‐based devices achieve the highest PCEs, reaching 18.71% and 18.69%, respectively, with pronounced improvements in *V_OC_* and FF relative to conventional HSLs. Extending this approach to ternary blends, devices based on PM6:BTP‐eC9:L8BO incorporating N‐SAM achieve a PCE of 20.15%, surpassing the corresponding binary devices through further enhancements in *V_OC_* and FF (Figure 6c). These results underscore the broad applicability and scalability of conjugated‐SAM‐based interfacial engineering for achieving simultaneously high efficiency and thermal stability in OSCs.

To quantitatively assess the impact of enhanced interfacial interactions on device operational stability under thermal stress, maximum power point tracking (MPPT) measurements were performed at 85°C using the binary PM6:BTP‐eC9 devices (Figure 6d, Figure S23). The T_90_ lifetime (defined as the time for the PCE to decay to 90% of its initial value) demonstrated a pronounced dependency on SAM conjugation, reaching 150(141.7 ± 7.4) h for N‐SAM‐based devices, compared with 48(44.9 ± 2.8) h for P‐SAM and only 9.5(8.68 ± 0.61) h for C‐SAM devices. This 15‐fold enhancement in thermal stability underscores the critical role of strong interfacial interactions in suppressing thermally induced morphological degradation, in agreement with the morphological analyses discussed above.

Temperature‐dependent MPPT measurements further elucidate the degradation behavior (Figure 6e and Figure S24). When the operating temperature is reduced to 65°C—representative of typical outdoor operating conditions under standard solar irradiation—the T_90_ lifetime of N‐SAM devices extends to 642 h. Analysis of the degradation kinetics, obtained by plotting the degradation rate (1/T_90_) as a function of inverse temperature, yields an activation energy (*E*a) of approximately 73 kJ mol^−^^1^ for the rate‐limiting degradation process (Figure 6f) [43, 44]. This activation energy provides quantitative insight into the underlying thermal degradation mechanism and enables predictive modeling of device lifetimes across a range of operating temperatures. Based on this Arrhenius analysis, N‐SAM‐based OSCs are projected to achieve a T_90_ lifetime of approximately 1609 h at room temperature (25°C) and a T_80_ lifetime exceeding 3218 h. These values represent conservative estimates, as device degradation is expected to transition to slower kinetics in the low‐temperature regime.

## 3 Conclusion

In conclusion, we demonstrate that rational extension of conjugation in SAM terminal groups enables tailored molecular interactions with both donor and acceptor components at the anode interface, providing a powerful strategy for interfacial engineering in OSCs. The enhanced π–π interactions and increased molecular rigidity of N‐SAM promote dense interfacial packing, favorable vertical phase distribution of BHJ film, and effective suppression of thermally induced morphological degradation. Consequently, N‐SAM delivers high PCEs of 19.73% in binary and 20.15% in ternary devices, together with a 15‐fold improvement in thermal operational stability (T_90_  =  150 h at 85°C). More broadly, this work establishes general molecular design principles highlighting how tailored interfacial interactions with both donor and acceptor materials can be leveraged to simultaneously optimize efficiency and durability, paving the way for the development of next‐generation interfacial structures for highly efficient and stable OSCs.

## Conflicts of Interest

The authors declare no conflicts of interest.

## Supporting information




**Supporting File 1**: adma73709‐sup‐0001‐Data1.xlsx.

**Supporting File 2**: adma73709‐sup‐0002‐Data2.xlsx.

**Supporting File 3**: adma73709‐sup‐0003‐SuppMat.docx.

## Data Availability

The data supporting this article have been included as part of the Supporting Information.
