# Supplementary material for: Strengthened Interfacial Coupling Between Self‐Assembled Monolayers and Bulk Heterojunctions Enables Thermally Stable Organic Solar Cells
Source: Adv Mater. 2026 Jun 14;38(40):e73709. doi: 10.1002/adma.73709 (PMC13378202; doi:10.1002/adma.73709)
Supplement: Supplementary file 3 — Supporting File 3: adma73709‐sup‐0003‐SuppMat.docx. [file ADMA-38-e73709-s001.docx]

Supporting Information

Strengthened Interfacial Coupling between Self-Assembled Monolayers and Bulk Heterojunctions Enables Thermally Stable Organic Solar Cells

*Gengxin Du,^1†^ Zhihong Wang,^1,2†^ Songyang Yuan,^3†^ Wenlin Jiang, ^1^ Shanchao Ouyang,^1^ Chengda Ge,^1^ Tian Xia, ^1^ Yiting Jiang, ^4^ Nan Zhang, ^1^ Yidan An, ^1^ Lingyi Ke, ^1^ Sai Wing Tsang,^1^ Francis R. Lin, ^1^ Qian Li,^1,5^ Alex K.-Y. Jen,^1,5,6,7^ Xuechen Jiao,^8^ Yong Zhang,^2*^ Hin-Lap Yip^1,4,5,7*^*

^1^Department of Materials Science and Engineering, City University of Hong Kong, Kowloon, Hong Kong 999077, China

^2^School of Materials Science and Engineering, Harbin Institute of Technology, Harbin 150001, China.

^3^School of Chemistry, Guangzhou Key Laboratory of Materials for Energy Conversion and Storage, Key Laboratory of Electronic Chemicals for Integrated Circuit Packaging, South China Normal University (SCNU), Guangzhou 510006, China

^4^School of Energy and Environmental Science, City University of Hong Kong, Kowloon, Hong Kong 999077, China

^5^Hong Kong Institute for Clean Energy (HKICE), City University of Hong Kong, Kowloon, Hong Kong 999077, China

^6^Department of Chemistry, City University of Hong Kong, Kowloon, Hong Kong 999077, China

^7^State Key Laboratory of Marine Environmental Health, City University of Hong Kong, Kowloon, Hong Kong 999077, China

^8^National synchrotron radiation laboratory, the University of Science of Technology of China, Hefei 230000, China

**Corresponding Author**

Hin-Lap Yip, E-mail: a.yip@cityu.edu.hk;

Yong Zhang, E-mail: yongzhang@hit.edu.cn.

**1. Materials**

All the reagents were used as received without any further purification. PM6 and BTP-eC9 were purchased from Solarmer. Isopropanol (IPA), toluene, methanol, ethanol, tetrahydrofuran (THF), and chloroform (CF) were purchased from Sigma-Aldrich. PNDIT-F3N was purchased from eFlexPV Limited.

**2. Synthesis of SAM**

For C-SAM:

**9-(4-bromophenyl)-9*H*-carbazole:** Carbazole (1 g, 0.00598 mol), 1-bromo-4-fluorobenzene (4 eq, 4.19 g, 0.02392 mol) and K_3_PO_4_ (3 eq, 3.81 g, 0.01794 mol) were combined in dry NMP (15 mL) under an argon atmosphere. The reaction mixture was stirred at 160 °C for 48 hours. Upon completion, the mixture was poured into brine (100 mL) and extracted with CH₂Cl₂ (3 × 80 mL). The combined organic extracts were concentrated under reduced pressure, and the crude product was purified by column chromatography (silica gel: 200-300 mesh; eluent: n-hexane/dichloromethane 4:1 (v/v)) to afford 1.60 g (65%) of the desired product as a white solid.


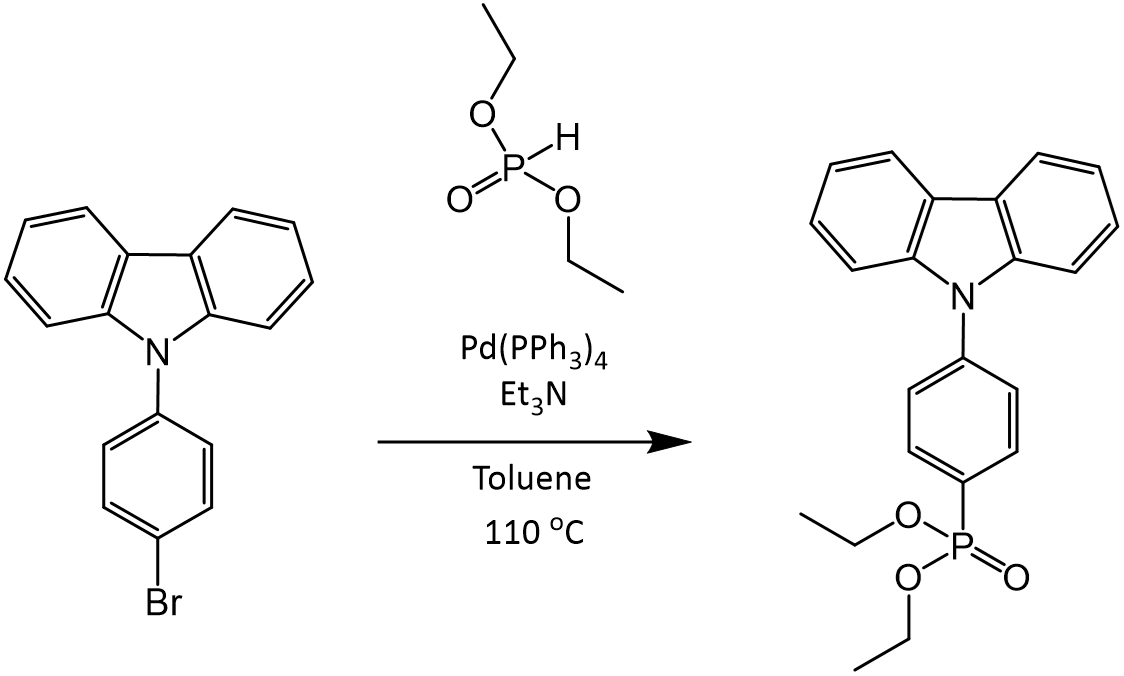


**Diethyl (4-(9H-carbazol-9-yl)phenyl)phosphonate:** 9-(4-bromophenyl)-9H-carbazole (1.20 g, 3.56 mmol), diethyl phosphite (1.5 eq, 738 mg, 5.34 mmol), Pd(PPh₃)₄ (5%, 206 mg, 0.178 mmol), triethylamine (8 mL) and toluene (40 mL) were charged into a 100 mL three-necked round-bottom flask. The mixture was heated to 110 ℃ and stirred under an argon atmosphere overnight. After the reaction was complete as monitored by TLC, the mixture was extracted with CH₂Cl₂ (3 × 50 mL). The combined organic extracts were dried over anhydrous sodium sulfate. Upon concentration under reduced pressure, the residue was purified by column chromatography (silica gel, using CH₂Cl₂/ethyl acetate = 4:1 (v/v) as eluent) to afford 1.02 g (75%) of the product as a white solid.


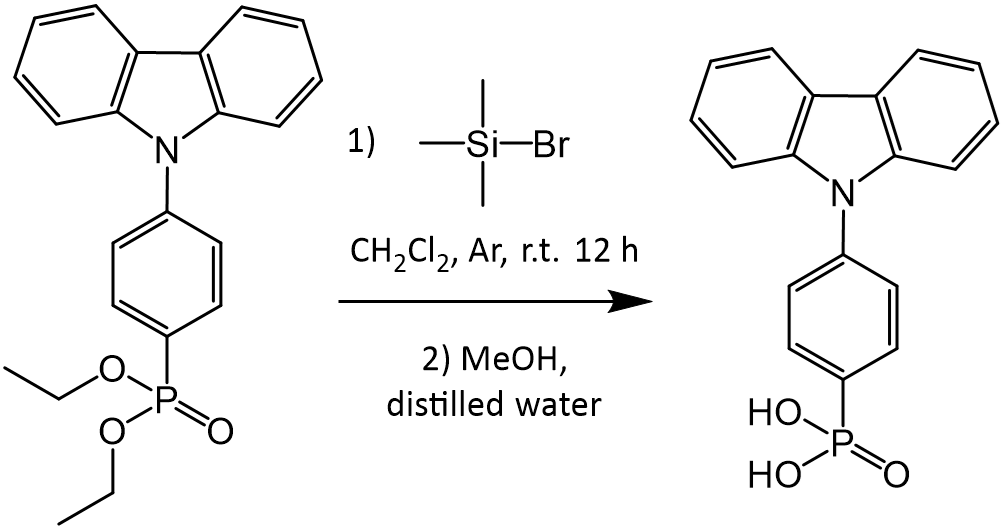


**(4-(9H-carbazol-9-yl)phenyl)phosphonic acid (C-SAM)**: Diethyl (4-(9H-carbazol-9-yl)phenyl)phosphonate (1 g, 2.28 mmol) was dissolved in anhydrous CH₂Cl₂ (20 mL) under an argon atmosphere. Bromotrimethylsilane (10 eq, 3.48 g, 2.9 mL, 22.8 mmol) was then added dropwise. The reaction mixture was stirred at room temperature for 12 hours under argon. Subsequently, the solvent was partially removed by distillation under reduced pressure. The resulting liquid residue was dissolved in methanol (6 mL), and distilled water (35 mL) was added dropwise until the solution turned opaque. The precipitate was collected by filtration and thoroughly washed with water to afford 0.70 g (82%) of the product as a white solid. (^1^H NMR (300 MHz, DMSO-*d*_6_) δ 8.26 (dd, *J* = 7.6, 1.1 Hz, 2H), 7.97 (dd, *J* = 12.6, 8.2 Hz, 2H), 7.75 (dd, *J* = 8.3, 2.9 Hz, 2H), 7.50 – 7.38 (m, 4H), 7.31 (dd, *J* = 8.0, 5.3, 2.8 Hz, 2H).  ^13^C NMR (75 MHz, DMSO-*d*_6_) δ 140.16, 139.55, 134.86, 133.05, 132.91, 126.89, 126.62, 126.43, 123.42, 121.10, 120.88, 110.17.)


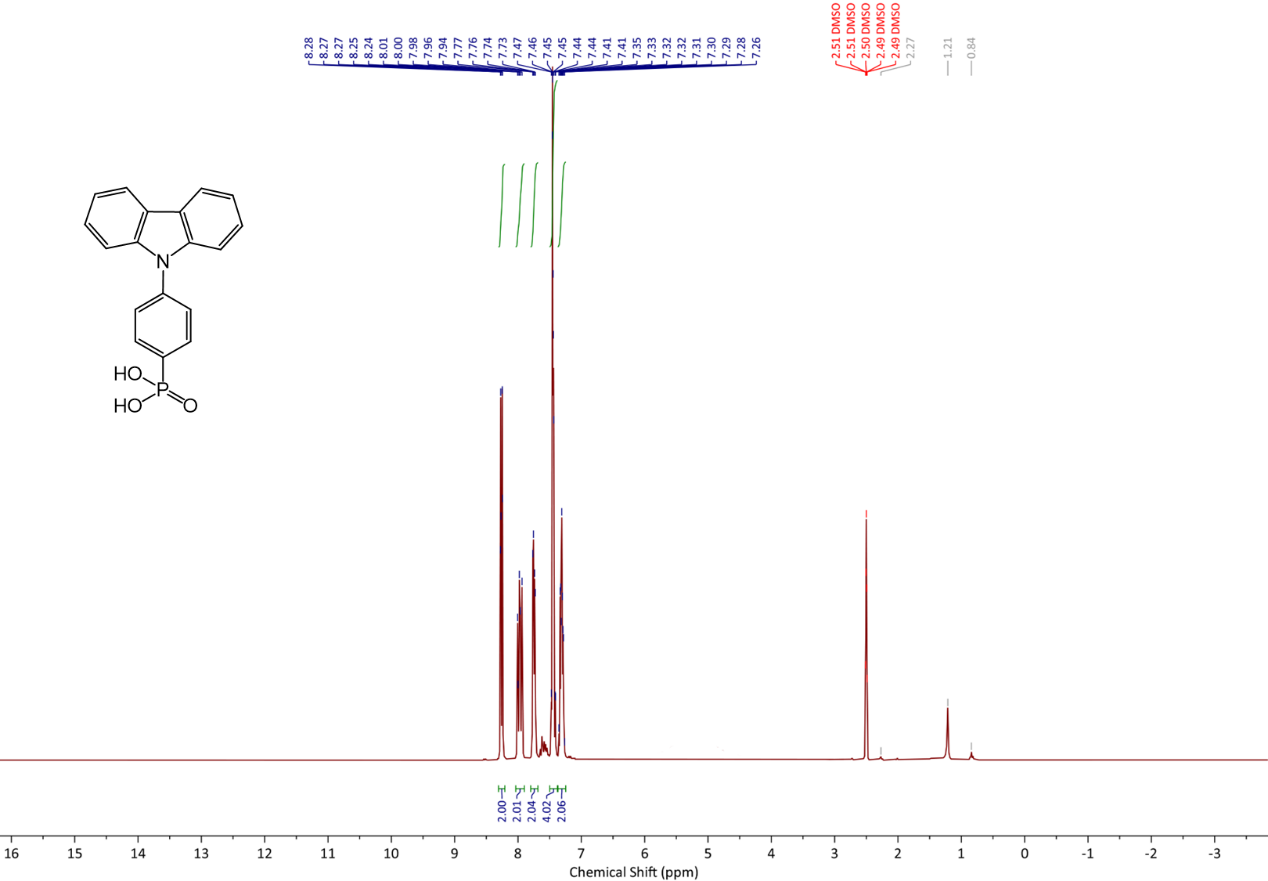


^1^H NMR spectra of (4-(9H-carbazol-9-yl)phenyl)phosphonic acid (**C-SAM**) in DMSO-*d*_6_.


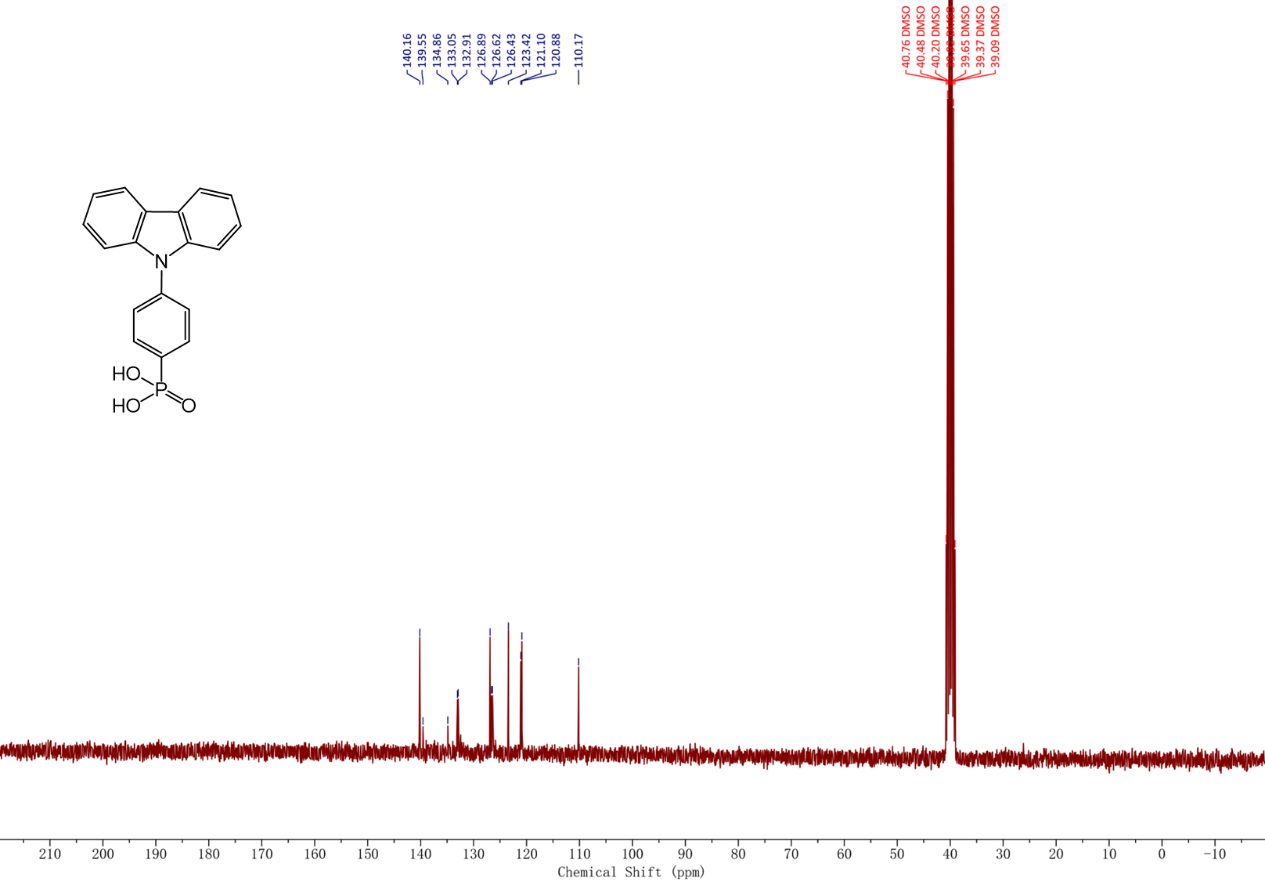


^13^C NMR spectra of (4-(9H-carbazol-9-yl)phenyl)phosphonic acid (**C-SAM**) in DMSO-*d*_6_.

For P-SAM:^1^

**9-(4-bromophenyl)-3,6-diphenyl-9*H*-carbazole:** A mixture of 3,6-diphenyl-9*H*-carbazole (1 g, 0.00313 mol), 1-bromo-4-fluorobenzene (6 eq, 3.29 g, 0.0188 mol), and K_3_PO_4_ (3 eq, 2 g, 0.0094 mol) in dry NMP (12 mL) was allowed to react under argon atmosphere. The reaction mixture was stirred for 40 h at 160 ^o^C. After reaction completion, the mixture was added to brine (100 mL) and extracted with CH_2_Cl_2_ (3 × 100 mL). The combined extract was concentrated under reduced pressure and the product was isolated by column chromatography (silica gel: 200–300 mesh; *n*-hexane: dichloromethane 4:1 (v/v) as eluent) to give 1.21 g (81 %) of white solid.

**Diethyl (4-(3,6-diphenyl-9*H*-carbazol-9-yl)phenyl)phosphonate:** 9-(4-bromophenyl)-3,6-diphenyl-9*H*-carbazole (1.69 g, 3.56 mmol), diethyl phosphite (1.5 eq, 738 mg, 5.34 mmol), Pd(PPh_3_)_4_ (5%, 206 mg, 0.178 mmol), triethylamine (8 mL) and toluene (40 mL) were added to a 100 mL three-necked round-bottom flask, heated and stirred at 110 ℃ under argon atmosphere overnight. After completion of the reaction (monitored by TLC), the reaction mixture was extracted repeatedly with CH_2_Cl_2_ for 3~4 times, and the organic phase was collected in a conical flask. The combined organic layers were dried over anhydrous Na_2_SO_4_. After concentration under vacuo, the residue was purified by column chromatography (silica gel, CH_2_Cl_2_: ethyl acetate = 4:1 (v/v) as eluent) to give 1.36 g (72 %) of white solid. ^1^H NMR (400 MHz, Chloroform-*d*) δ 8.41 (d, *J* = 1.7 Hz, 2H), 8.10 (dd, *J* = 12.9, 8.2 Hz, 2H), 7.80 – 7.67 (m, 8H), 7.56 (d, *J* = 8.5 Hz, 2H), 7.50 (t, *J* = 7.7 Hz, 4H), 7.41 – 7.34 (m, 2H), 4.34 – 4.18 (m, 4H), 1.43 (t, *J* = 7.1 Hz, 6H). ^13^C NMR (100 MHz, Chloroform-*d*) δ 141.67, 140.13, 134.31, 133.72, 133.61, 128.88, 128.27, 127.34, 126.81, 126.50, 126.37, 126.35, 125.91, 124.49, 119.02, 110.17, 62.49, 62.44, 16.52, 16.46.

**(4-(3,6-diphenyl-9*H*-carbazol-9-yl)phenyl)phosphonic acid (P-SAM):** Diethyl (4-(3,6-diphenyl-9*H*-carbazol-9-yl)phenyl)phosphonate (1 g, 1.88 mmol) was dissolved in anhydrous CH_2_Cl_2_ (24 ml) under argon atmosphere and bromotrimethylsilane (10 eq, 2.88 g 2.5 mL, 18.8 mmol) was added dropwise. The reaction was stirred for 12 h at room temperature under an argon atmosphere. Afterward, the solvent was partially distilled off under reduced pressure, and the liquid residue was dissolved in methanol (5 ml). Next, distilled water was added dropwise (40 ml), until the solution became opaque. The product was filtered off and washed with water to give 0.72 g (80 %) of white solid. ^1^H NMR (400 MHz, DMSO-*d*_6_) δ 8.76 (d, *J* = 1.8 Hz, 2H), 8.04 – 7.97 (m, 2H), 7.87 – 7.76 (m, 8H), 7.58 – 7.47 (m, 6H), 7.40 – 7.32 (m, 2H). ^13^C NMR (151 MHz, DMSO-d6) δ 141.11, 140.20, 139.48, 134.39, 133.38, 133.07, 133.00, 129.41, 127.28, 127.19, 126.43, 126.34, 125.96, 124.42, 119.56, 110.75, 2.50, 2.30.


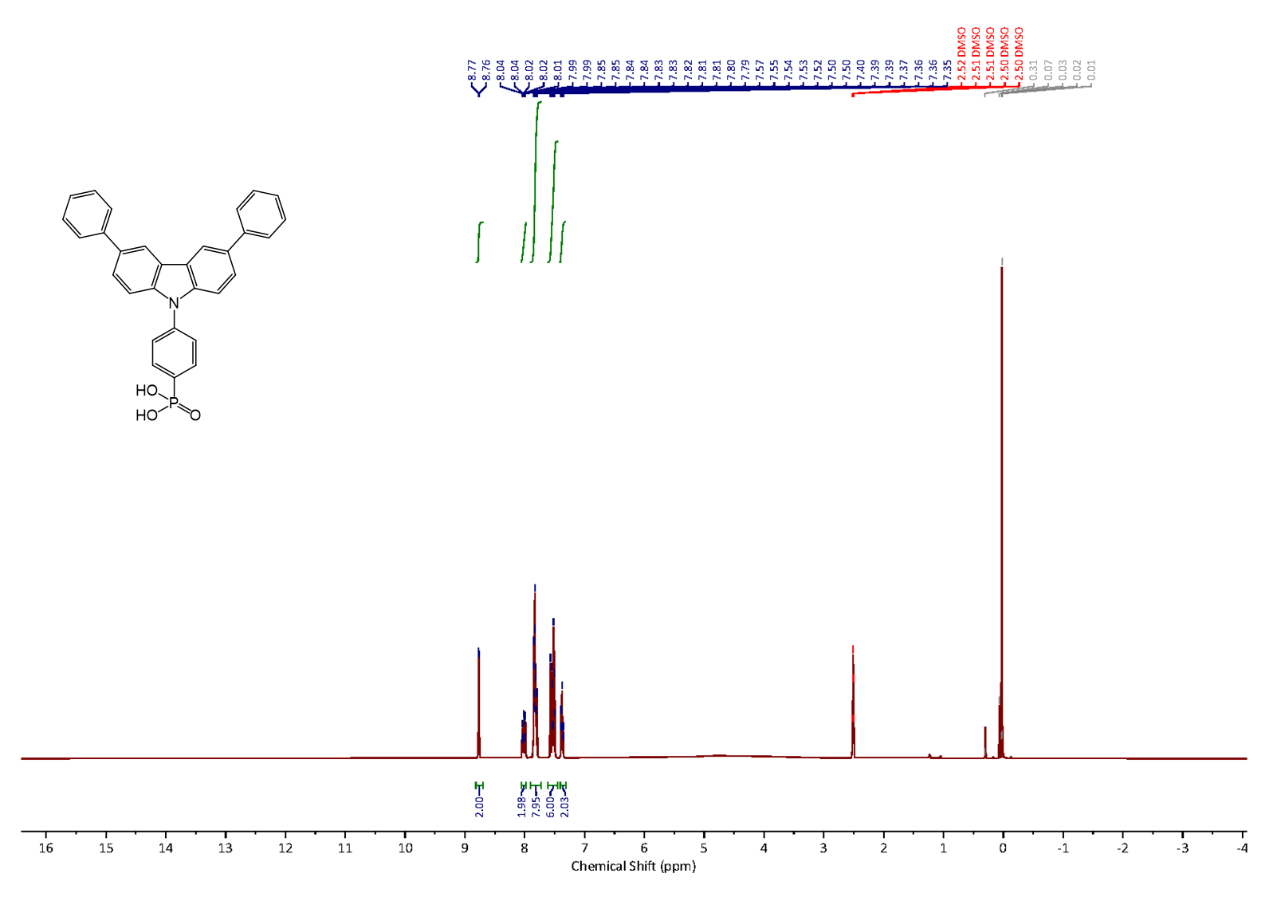


^1^H NMR spectra of (4-(3,6-diphenyl-9*H*-carbazol-9-yl)phenyl)phosphonic acid (**P-SAM**) in DMSO-*d*_6_.


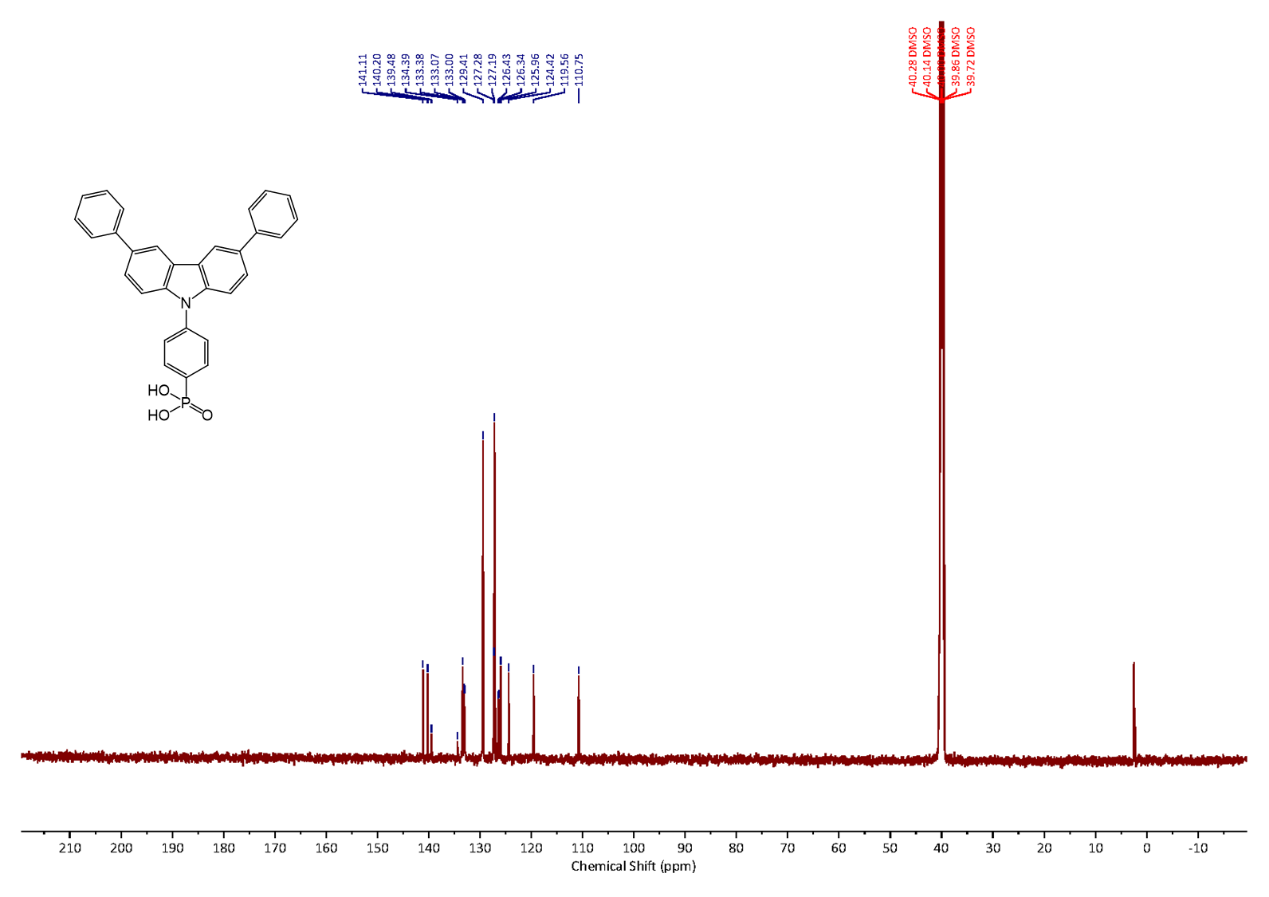


^13^C NMR spectra of (4-(3,6-diphenyl-9*H*-carbazol-9-yl)phenyl)phosphonic acid (**P-SAM**) in DMSO-*d*_6_.

For N-SAM:

**3,6-di(naphthalen-2-yl)-9*H*-carbazole**: A mixture of 3,6-dibromo-9H-carbazole (1.00 g, 3.09 mmol), 2-bromonaphthalene (2.2 eq, 1.56 g, 6.80 mmol), Pd(PPh₃)₄ (5 mol%, 178 mg, 0.155 mmol), and potassium carbonate (3 eq, 1.28 g, 9.27 mmol) in toluene/ethanol (30 mL, 4:1 v/v) was heated to 110 °C and stirred under an argon atmosphere for 24 hours. After the reaction was complete (monitored by TLC), the mixture was cooled to room temperature and poured into brine (80 mL). The aqueous layer was extracted with dichloromethane (3 × 50 mL). The combined organic extracts were dried over anhydrous Na₂SO₄ and concentrated under reduced pressure. The crude product was purified by column chromatography (silica gel, eluting with n-hexane/dichloromethane 3:1 v/v) to afford 1.15 g (70%) of the product as a light yellow solid.


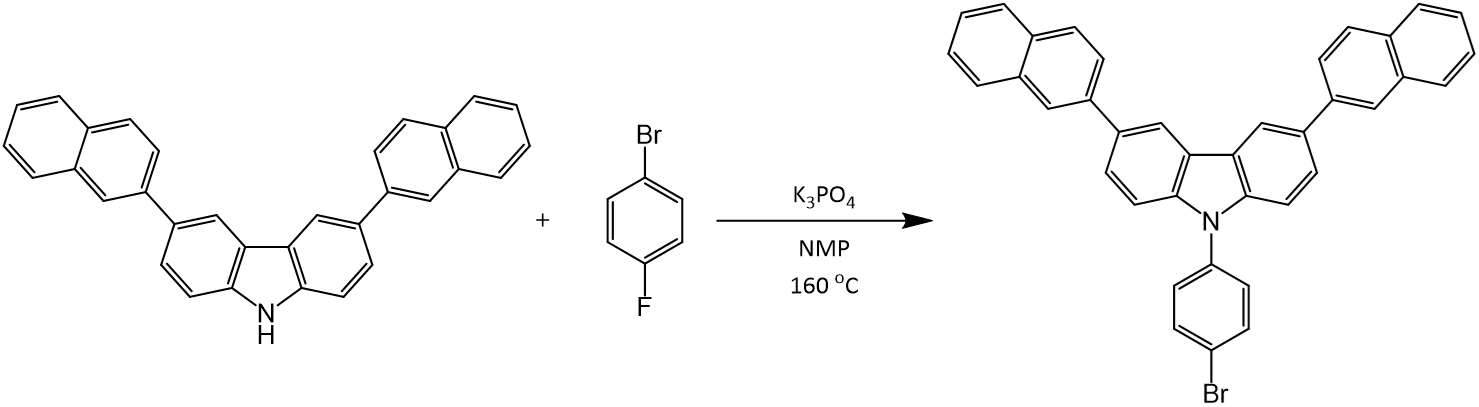


**9-(4-bromophenyl)-3,6-di(naphthalen-2-yl)-9H-carbazole**: A mixture of 3,6-di(naphthalen-2-yl)-9H-carbazole (1 g, 0.00227 mol), 1-bromo-4-fluorobenzene (6 eq, 2.38 g, 0.0136 mol), and K₃PO₄ (3 eq, 0.94 g, 0.00681 mol) in dry NMP (12 mL) was allowed to react under an argon atmosphere. The reaction mixture was stirred for 40 h at 160 °C. After reaction completion, the mixture was added to brine (100 mL) and extracted with CH₂Cl₂ (3 × 100 mL). The combined organic extract was concentrated under reduced pressure and the product was isolated by column chromatography (silica gel: 200–300 mesh; n-hexane/dichloromethane 4:1 (v/v) as eluent) to give 0.75 g (75%) of a white solid.


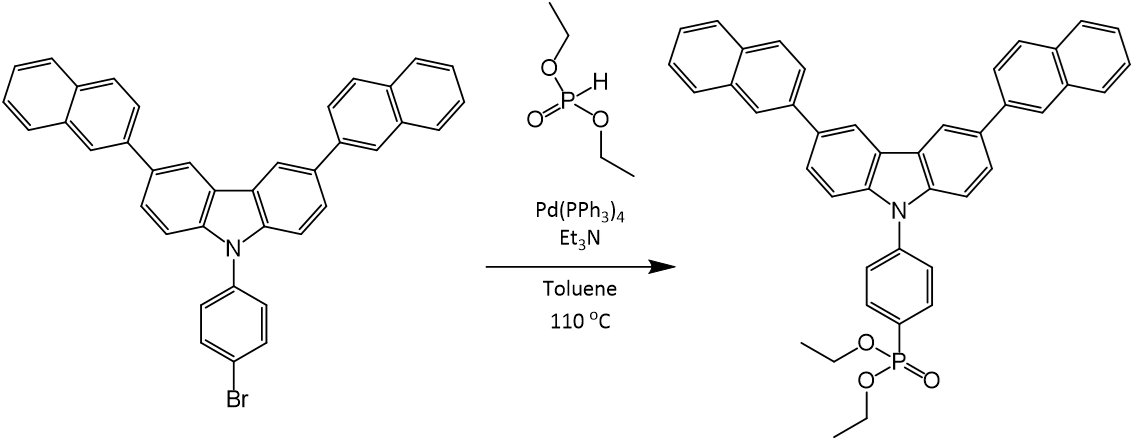


**Diethyl (4-(3,6-di(naphthalen-2-yl)-9H-carbazol-9-yl)phenyl)phosphonate**: A mixture of 9-(4-bromophenyl)-3,6-di(naphthalen-2-yl)-9H-carbazole (1.00 g, 1.74 mmol), diethyl phosphite (1.5 eq, 0.36 g, 2.61 mmol), Pd(PPh₃)₄ (5 mol%, 100 mg, 0.087 mmol), triethylamine (8 mL) and toluene (40 mL) was added to a 100 mL three-necked round-bottom flask. The reaction mixture was heated to 110 ℃ and stirred under an argon atmosphere overnight. After completion of the reaction (monitored by TLC), the mixture was extracted with CH₂Cl₂ (3 × 50 mL). The combined organic layers were dried over anhydrous Na₂SO₄. After concentration under reduced pressure, the residue was purified by column chromatography (silica gel, CH₂Cl₂/ethyl acetate = 4:1 (v/v) as eluent) to give 0.83 g (70%) of the product as a white solid.


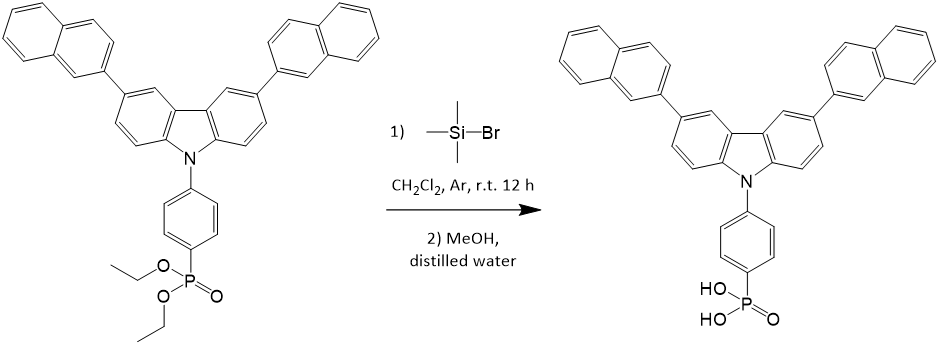


**(4-(3,6-di(naphthalen-2-yl)-9H-carbazol-9-yl)phenyl)phosphonic acid**: Diethyl (4-(3,6-di(naphthalen-2-yl)-9H-carbazol-9-yl)phenyl)phosphonate (1 g, 1.52 mmol) was dissolved in anhydrous CH₂Cl₂ (20 mL) under an argon atmosphere, and bromotrimethylsilane (10 eq, 2.33 g, 2.0 mL, 15.2 mmol) was added dropwise. The reaction was stirred for 12 h at room temperature under an argon atmosphere. Afterward, the solvent was partially distilled off under reduced pressure, and the liquid residue was dissolved in methanol (5 mL). Next, distilled water (40 mL) was added dropwise until the solution became opaque. The product was filtered off and washed with water to give 0.76 g (80%) of a white solid. ^1^H NMR (400 MHz, DMSO-*d*_6_) δ 8.97 (d, J = 1.9 Hz, 1H), 8.39 (s, 1H), 8.09 – 7.94 (m, 6H), 7.86 (dd, J = 8.4, 2.8 Hz, 1H), 7.67 – 7.49 (m, 4H). ^13^C NMR (151 MHz, DMSO-*d*_6_) δ 140.36, 139.41, 138.45, 134.02, 133.13, 133.04, 132.46, 131.99, 128.91, 128.52, 128.02, 126.89, 126.48, 126.30, 125.92, 125.28, 124.56, 119.90, 110.90.


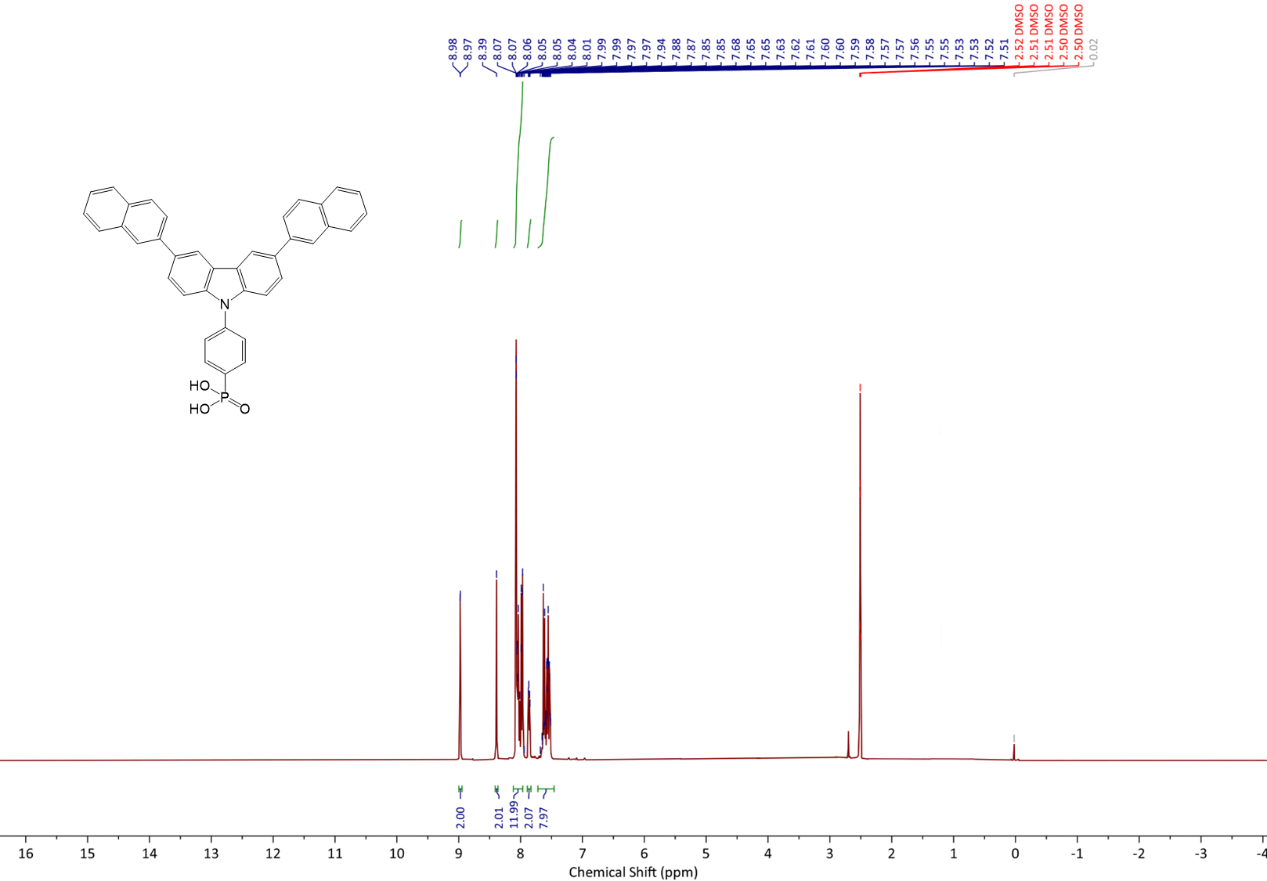


^1^H NMR spectra of (4-(3,6-di(naphthalen-2-yl)-9H-carbazol-9-yl) phenyl) phosphonic acid (**N-SAM**) in DMSO-*d*_6_.


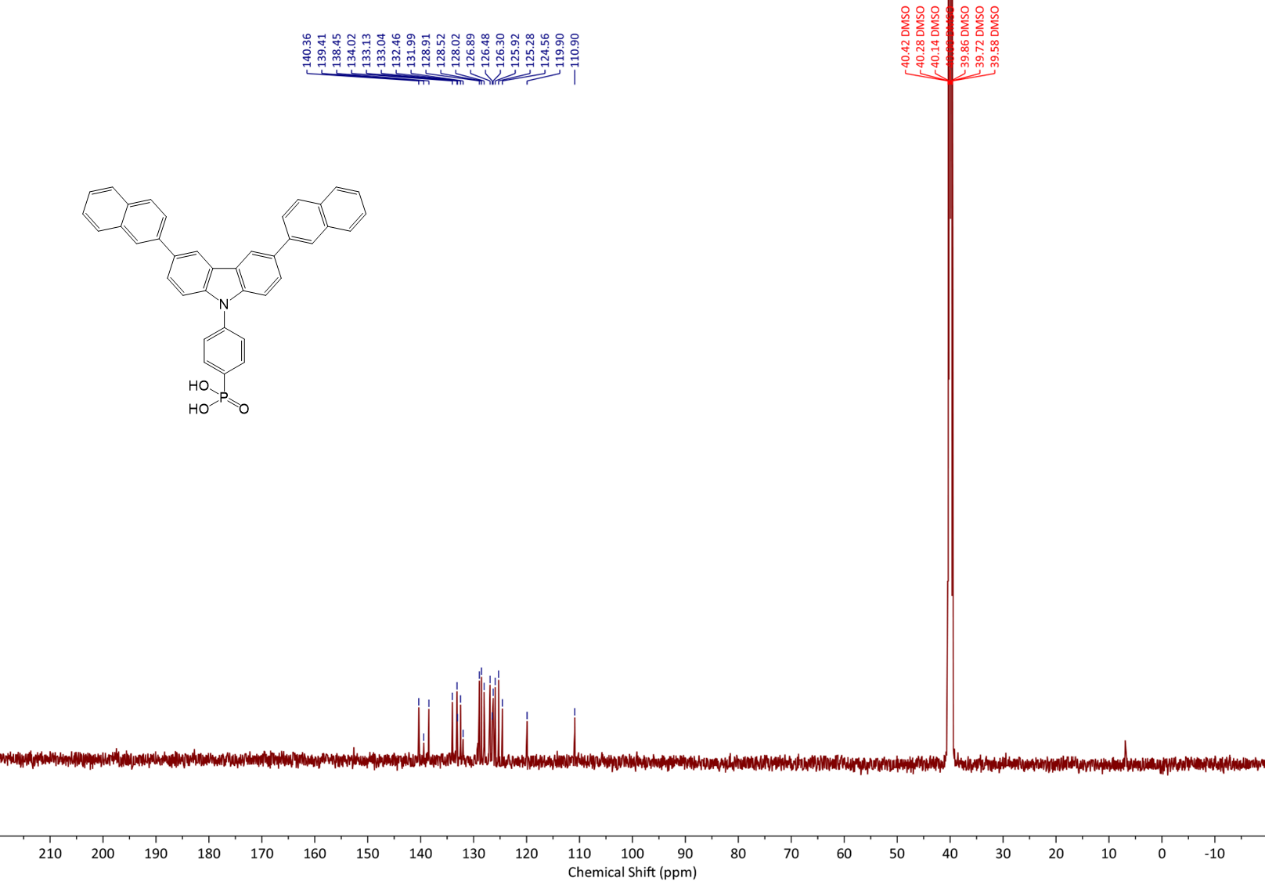


^13^C NMR spectra of (4-(3,6-di(naphthalen-2-yl)-9H-carbazol-9-yl) phenyl) phosphonic acid (**N-SAM**) in DMSO-*d*_6_.

**3. Device fabrication**

The ITO substrates were cleaned by ultrasonication sequentially with dilute detergent solution, deionized water, acetone, and isopropanol for 20 min each step before being dried in an oven setting @ 80 °C. The substrates were treated with UV-ozone for 20 min before use. The SAM solutions were prepared by dissolving SAM powder in THF, azeotrope of methanol/THF (with a mole fraction of 0.492:0.508),^2^ or azeotrope of ethanol/THF (with a mole fraction of 0.858:0.142)^3^ at 1 mg/ml concentration and stirred for 2 hours before use. All SAMs printed by slot-die coating platform were performed in ambient conditions. The printing velocity, gap between coating head and substrate, flow rate, and substrate temperature for SAM were adjusted to 25 mm/s, 100 μm, 5 μL/min, and 65 °C. After annealing at 100 °C for 10 min, the substrate was transferred into the glove box for temporary storage. PM6: BTP-eC9 (1:1.2) or PM6:BTP-eC9:L8BO-2F(1:0.96:0.24) blend was dissolved in chloroform with a concentration of 6.25 mg/ml of PM6 for spin coating. The solution was stirred at 50 °C for at least 1h. For the spin-coated active layer, the rotation speed is 2600 rpm. After coating, the active layer was annealed at 100 °C for 5 min. PNDIT-F3N was dissolved in methanol (with 0.5 vt% acetic acid) with 0.5 mg/ml concentration for spin coating. For spin-coated PNDIT-F3N, the rotation speed is 1800 rpm. Then, samples were transferred to the evaporation chamber for the deposition of Ag (100 nm). The OSCs have an identical active area of 0.04 cm^2^ defined by the overlap area of the anode and the cathode. For *J*-*V* performance measurement, a test mask with an accurate area of 0.0324 cm^2^ was used.

**4. Instruments and Characterizations**

Dynamic light scattering (DLS) experiments were conducted by a dynamic light scattering particle size Analyzer (Malven Zeta sizer Nano ZS) at 25 °C with a monochromatic coherent He–Ne laser (640 nm) as the light source. An avalanche photodiode detector that detected the scattered light at an angle of 173°. DLS measurements were carried out in SAM solutions in THF, azeotrope of methanol/THF, or azeotrope of ethanol/THF to determine the size of the particles. Contact angle was measured with a DataPhysics contact angle tester and the water drop volume was set as 3 μL. The cyclic voltammetry experiments were performed at room temperature in a nitrogen atmosphere with a three-electrode system using a bare ITO or SAM-modified ITO as the working electrode, Pt wire as the counter electrode, and an Ag/AgCl (saturated KCl) as the reference electrode. Tetrabutylammonium phosphorus hexafluoride (Bu_4_NPF_6_, 0.1M) in *o*-DCB solution was used as the supporting electrolyte, and a series of scan rates was applied. For calibration, the redox potential of ferrocene/ferrocenium (Fc/Fc+) was measured under the same condition. The detailed calculation for the surface density of SAM molecules on ITO can be found in literature.^1^

AFM and KPFM images were probed by a Dimension Icon AFM (Bruker) with the tapping mode at ambient conditions. UV-vis absorption spectra were characterized by a Hitachi UH4150 UV-VIS-NIR Spectrophotometer. Dynamic MPP (Maximum power point) tracking was recorded on an in-situ stability measurement system (CRYSCO, Guangzhou) with a white LED lamp light source. The testing devices (0.04 cm^2^) based on PM6:BTP-eC9 system were unencapsulated and put in the sample chamber, which was placed in the nitrogen-filled glove box. The UPS and XPS characterizations were performed by a VG ESCALAB 220i-XL surface analysis system equipped with a He discharge lamp (hv = 21.22 eV) and a monochromatic Al– Kα X-ray gun (hv = 1486.6 eV). The SAM solutions were deposited on ITO in the same process as device fabrication. Typically, the characterized peak of hydrocarbon C1s from adventitious carbon at 284.8 eV was used for binding energy calibration.

The *J-V* characteristics of the OSC devices were measured under a solar simulator (Enlitech, SS-F5, Taiwan) using a Keithley 2400 source meter in a nitrogen glove box at room temperature. The light intensity is calibrated using KG2 NREL-calibrated silicon solar cells, giving a value of 100 mW cm^−2^. EQE spectra are measured by EnLi Technology (Taiwan) EQE measurement system equipped with a standard silicon diode, where the monochromatic light was generated from a Newport 300 W lamp.

Resonant soft X-ray scattering (RSoXS): The RsoXS experiment was performed at Hefei Light Source Soft X-ray Resonant Scattering Beam Station (BL05U-B). Blend films for the measurement were prepared with a ~100 nm thickness on top of ITO/PEDOT:PSS/ZnO/SAMs (PEDOT:PSS is used to remove the top blend film, and ZnO is prepared by atomic layer deposition technology to provide a thin layer (~3 nm) of anchoring substrate for SAMs). The sample was placed on a 1.0 mm × 1.0 mm Si_3_N_4_ membrane supported by a 200-μm thick, 5 mm × 5 mm silicon frame.

Photoluminescence Quantum Yield (PLQY): PLQY calculations are based on a home-made experimental platform, mainly including a 640 nm laser source, an integrating sphere, and a spectrometer. Spectra were acquired under three conditions: no sample (experiment a), indirect laser incidence on the sample (experiment b), and direct laser incidence on the sample (experiment c).^4^ The integrated intensity was calculated using the method provided in and substituted into the equation 1 for the calculation:

$PLQY=\frac{P_{c}-(1-A)\times P_{b}}{L_{a}\times A}\times100\%$ (1)

A is the absorption, A= 1-L_c_/L_b_. L_a_, L_b_ and L_c_ is the integated light intensity in experiment a, b and c. P_b_ and P_c_ is the integrated PL intensity as shown in Figure S12c and d.

**5. Computational methods**

Molecular dynamics simulations are carried out to investigate the adsorption distribution of the PM6 and BTP-eC9 molecules near the confined SAM layer from the atomic level. The SAM substrates will first undergo a thorough structural optimization and then set as rigid to ensure that the atoms of the SAM layer are fixed during the simulation. First, a geometry optimization is employed to relax the simulation box. Then, a canonical (NVT) ensemble with a 1.0 fs time step is employed to optimized the simulation box, where the temperature is set to 300 K. The temperature is kept via the Nose-Hoover thermostat. The optimization time is set to 5.0 ns, which is long enough to obtain a stable system. In all the MD simulation, the motion of atoms is described by classical Newton’s equation, which is solved using the velocity-Verlet algorithm. The packing of the three different SAMs was also optimized structurally using a similar method. It is worth noting that when constructing the model, there were 5 molecules involved in the packing of the SAMs, in order to fully consider the possible different configurations of the SAMs during the packing process.

Density functional theory (DFT) calculations were carried out using the CP2K v2024.1 software package. A double-zeta valence polarized basis set (DZVP-MOLOPT-SR-GTH) was employed along with the Perdew–Burke–Ernzerhof (PBE) exchange–correlation functional ^5, 6^ The Quickstep method was used to perform single-point energy calculations under periodic boundary conditions in all three directions. The plane-wave energy cutoff was set to 450 Ry for the electron density expansion. The convergence criteria for the inner self-consistent field (SCF) cycle were set to 5*10^-6^ eV for the total energy and 0.05 eV Å^−1^ or the maximum atomic force.

For the binding energy calculation, the binding energy (${\Delta E}_{binding}$) is defined as:

${\Delta E}_{binding}=E_{slab+molecule}-E_{slab}-E_{molecule}$ (2)

where $E_{slab}$ and $E_{molecule}$ are the total energy of the slab and adsorbed SAM molecule, respectively.

The adsorption energy E_ads_ was calculated as:

E_ads_=E_total_−E_slab_−E_adsorbate_  (3)

in which E_total_ represents the total energy of the adsorption system, and E_adsorbate_ is the energy of the isolated adsorbate. To ensure consistency, the isolated adsorbate energy was computed using the same computational parameters and k-point sampling as those applied in the adsorption system.

The non-covalent interaction is systematically computed to characterize the spatial distribution of non-covalent bonding interactions (Van der Waals' force and hydrogen bonding interactions e.g.) within the system. All calculations were performed within the framework of DFT calculation, with wavefunction data extracted from Self-consistent field (SCF) converged charge densities. The date post-processing and visualizing were used the Multiwfn 3.8 and VMD (Visual Molecular Dynamics) 1.9.4 package, respectively.^7-9^

**6. Supporting Figures and Tables**


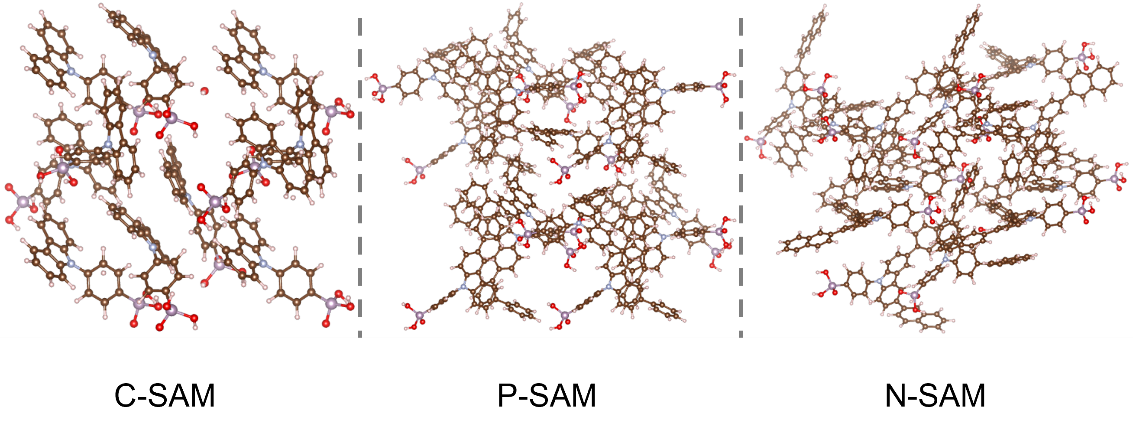


Figure S1. Theoretical calculation of the configurations of C-SAM, P-SAM, and N-SAM in free space.


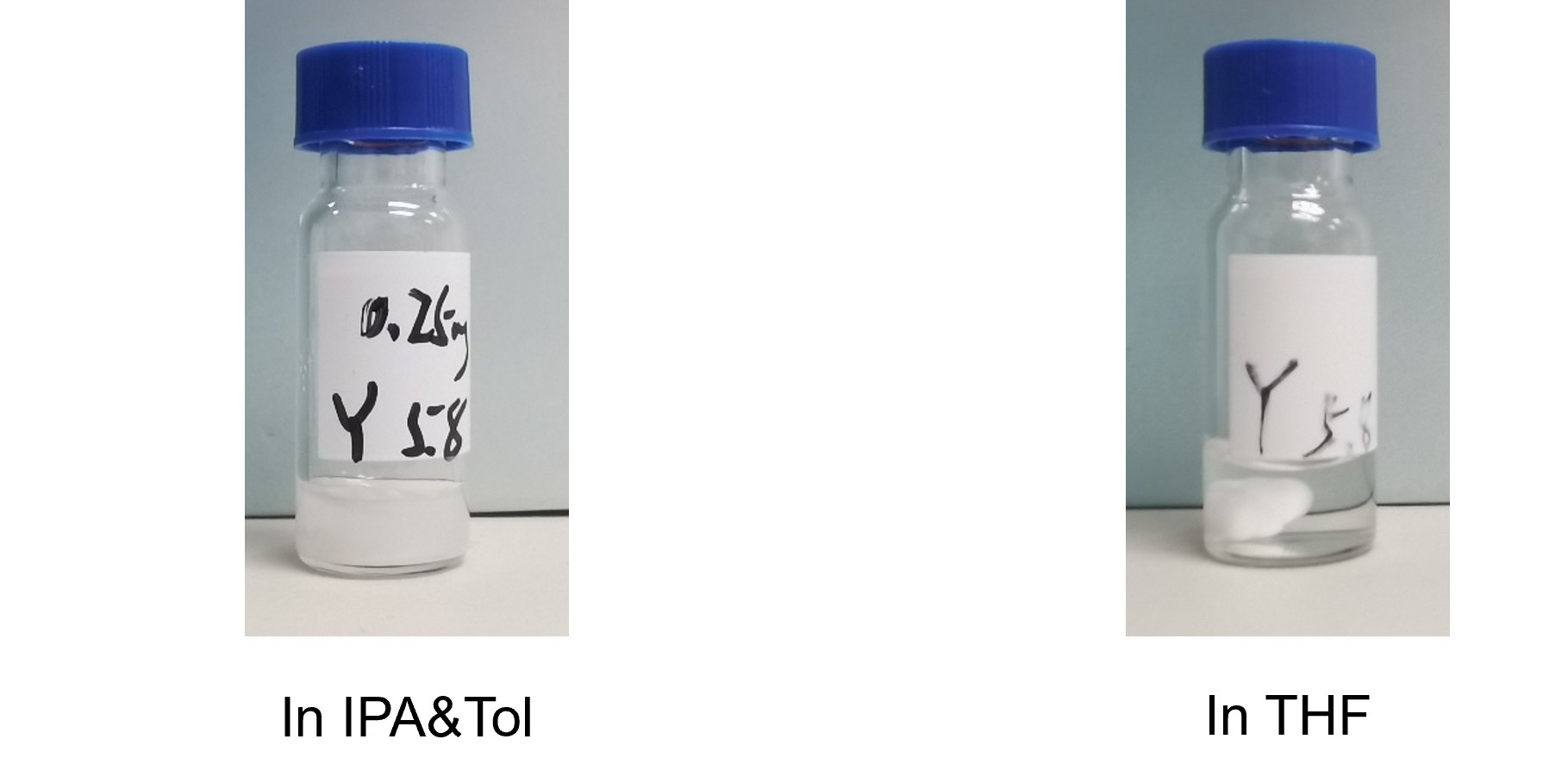


Figure S2. Dispersion of N-SAM molecules in azeotrope of IPA and toluene (IPA&Tol, left) and tetrahydrofuran (THF, right).


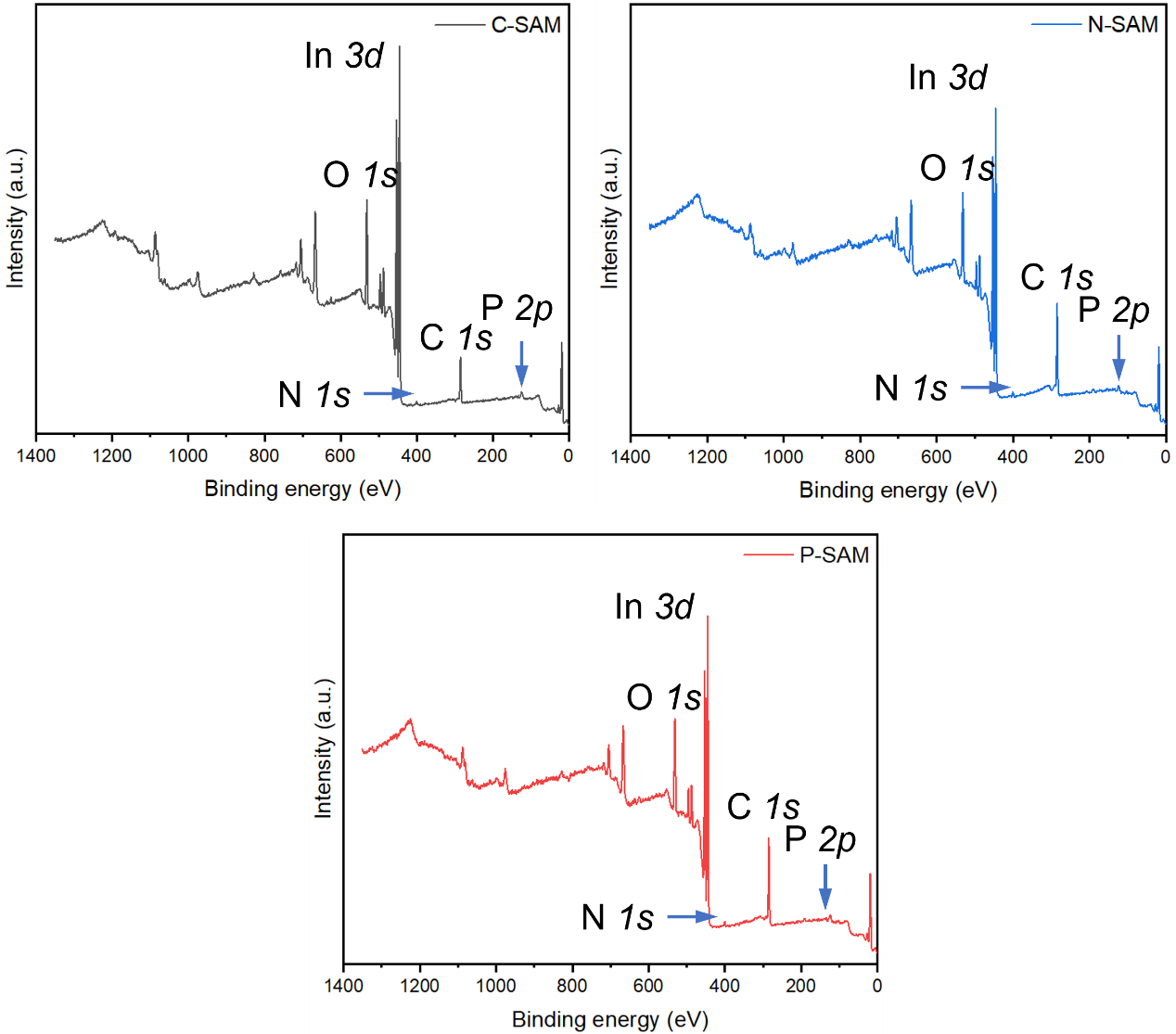


Figure S3. XPS spectra of different deposited SAMs on the ITO substrate.


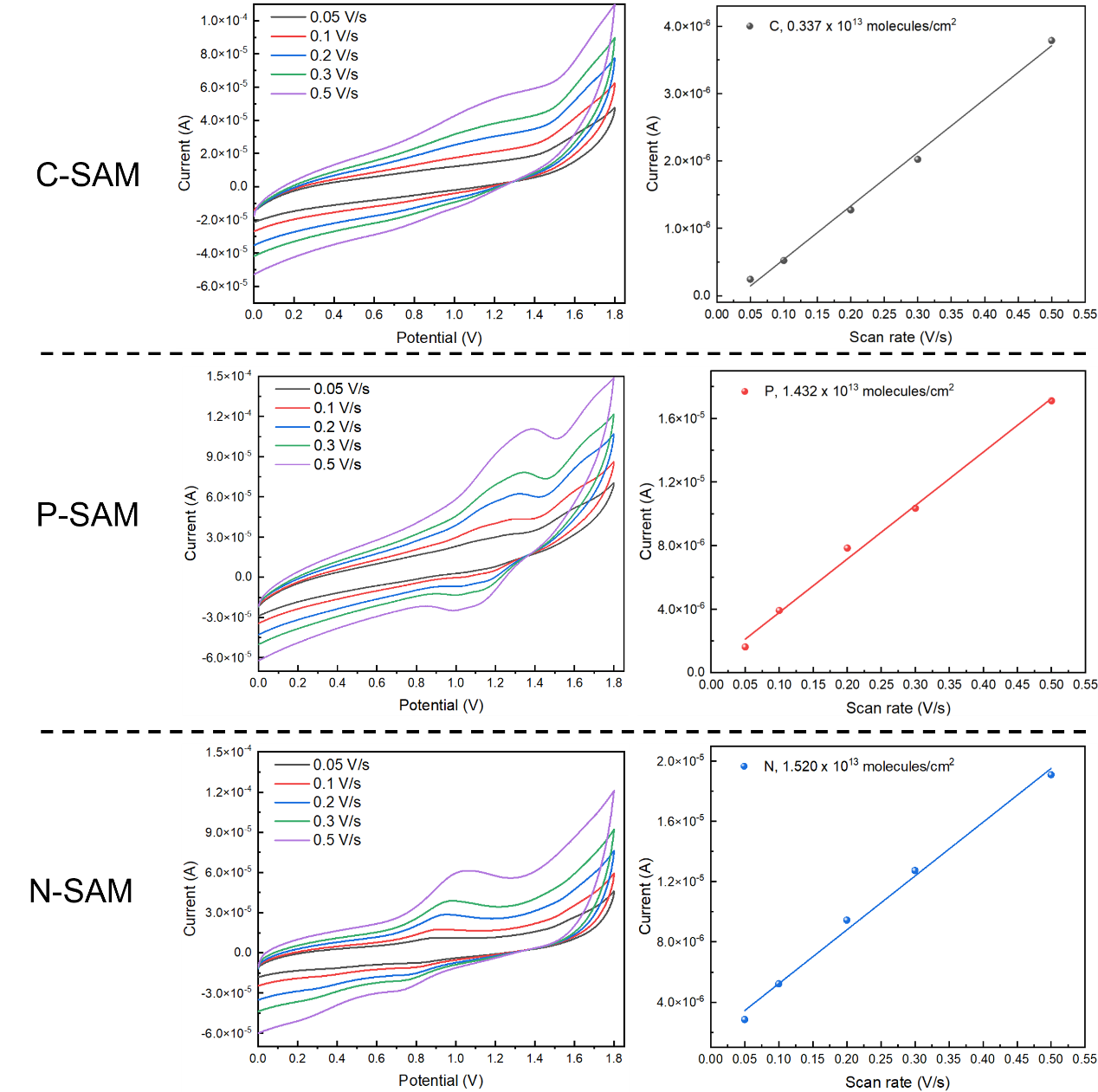


Figure S4. Cyclic voltammetry curves of different ITO/SAMs substrates, and the surface density of SAMs obtained based on the corresponding peak current-scan rate relationship.


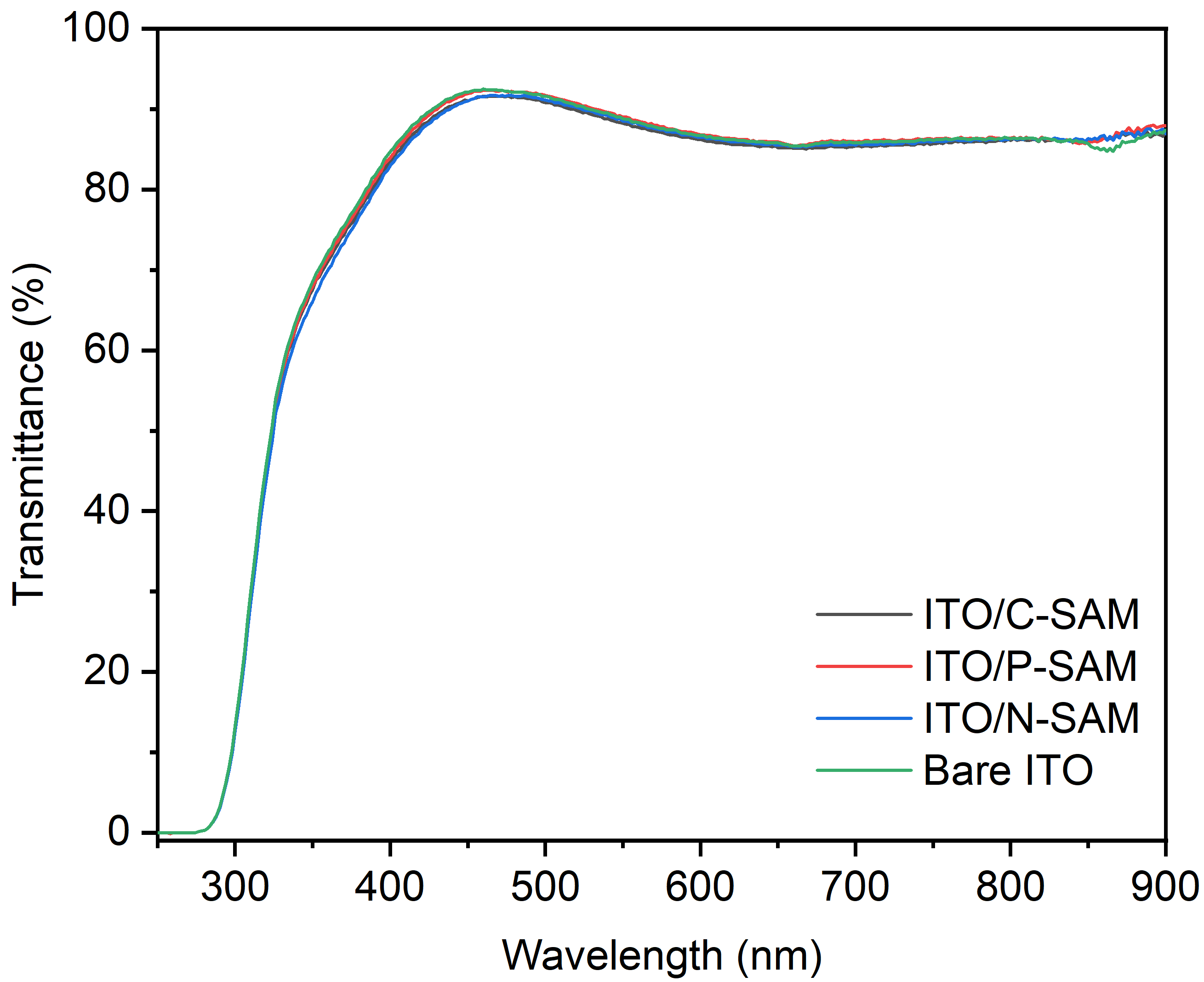


Figure S5. Transmission spectra of bare ITO and different SAMs coated ITO.


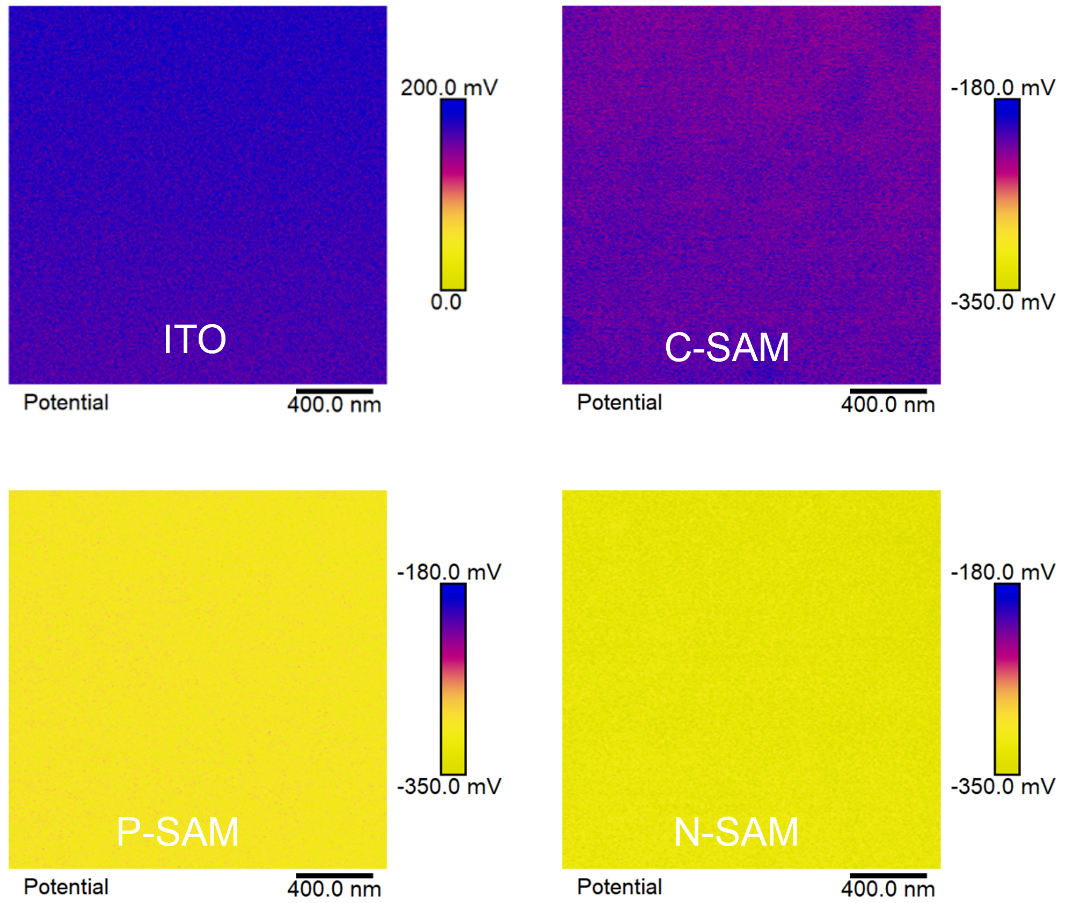


Figure S6. KPFM potential images of bare ITO and different SAMs modified ITO substrates.


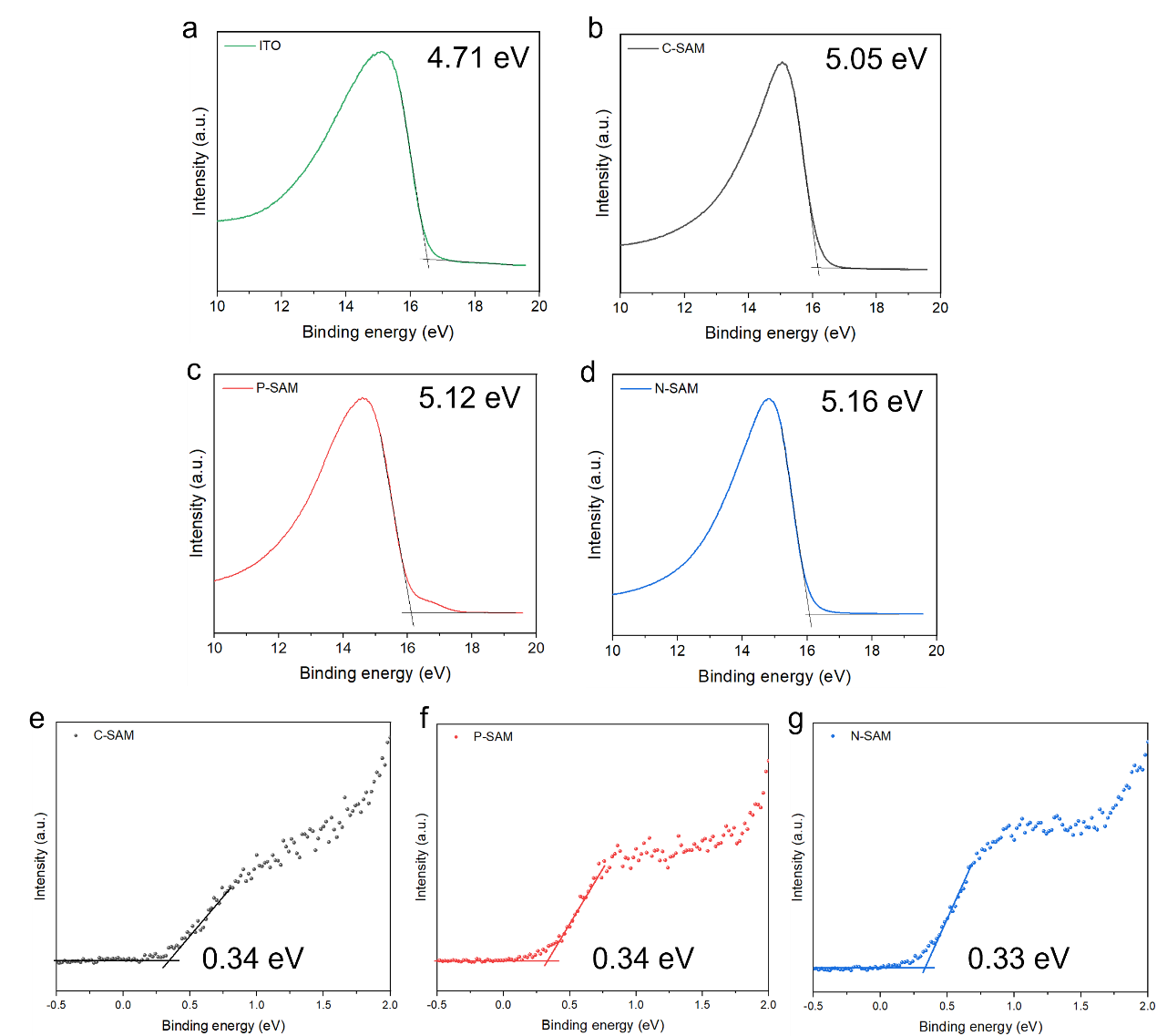


Figure S7. UPS spectra for work function (WF) extraction, performed by using: WF = 21.22 eV-*E*_cut-off_ (a-d): bare ITO (a), C-SAM modified ITO (b), P-SAM modified ITO (c), N-SAM modified ITO (d); UPS spectra for and *E*_∆_ extraction (e-g): C-SAM modified ITO (e), P-SAM modified ITO (f), N-SAM modified ITO (g).


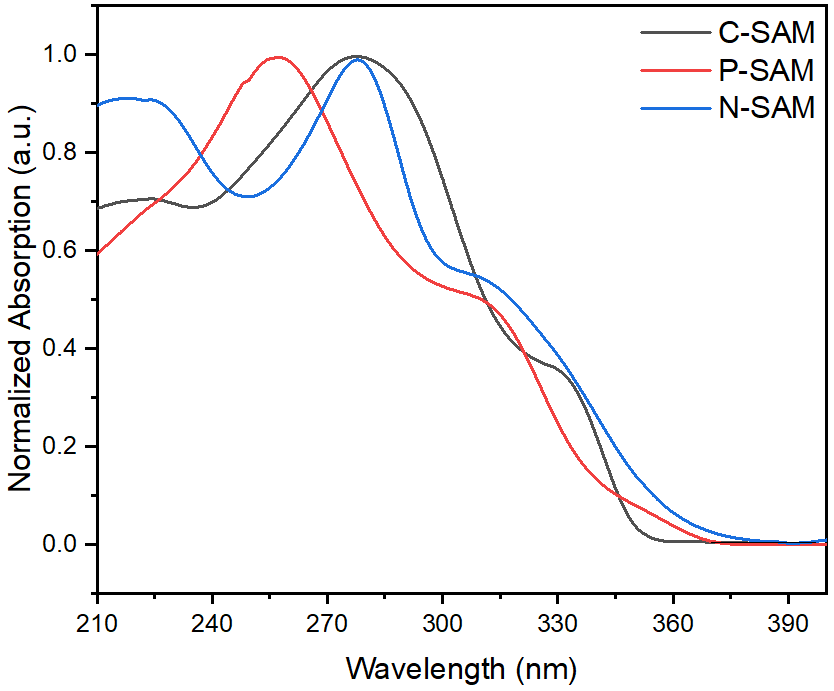


Figure S8. Band gap extracted based on the absorption edge position of the SAMs in solution: 3.55 eV (C-SAM), 3.36 eV (P-SAM), 3.43 eV (N-SAM). The estimated LUMO levels are: -1.84 eV (C-SAM), -2.10 eV (P-SAM), and -2.06 eV (N-SAM), by using band gap = LUMO-HOMO.
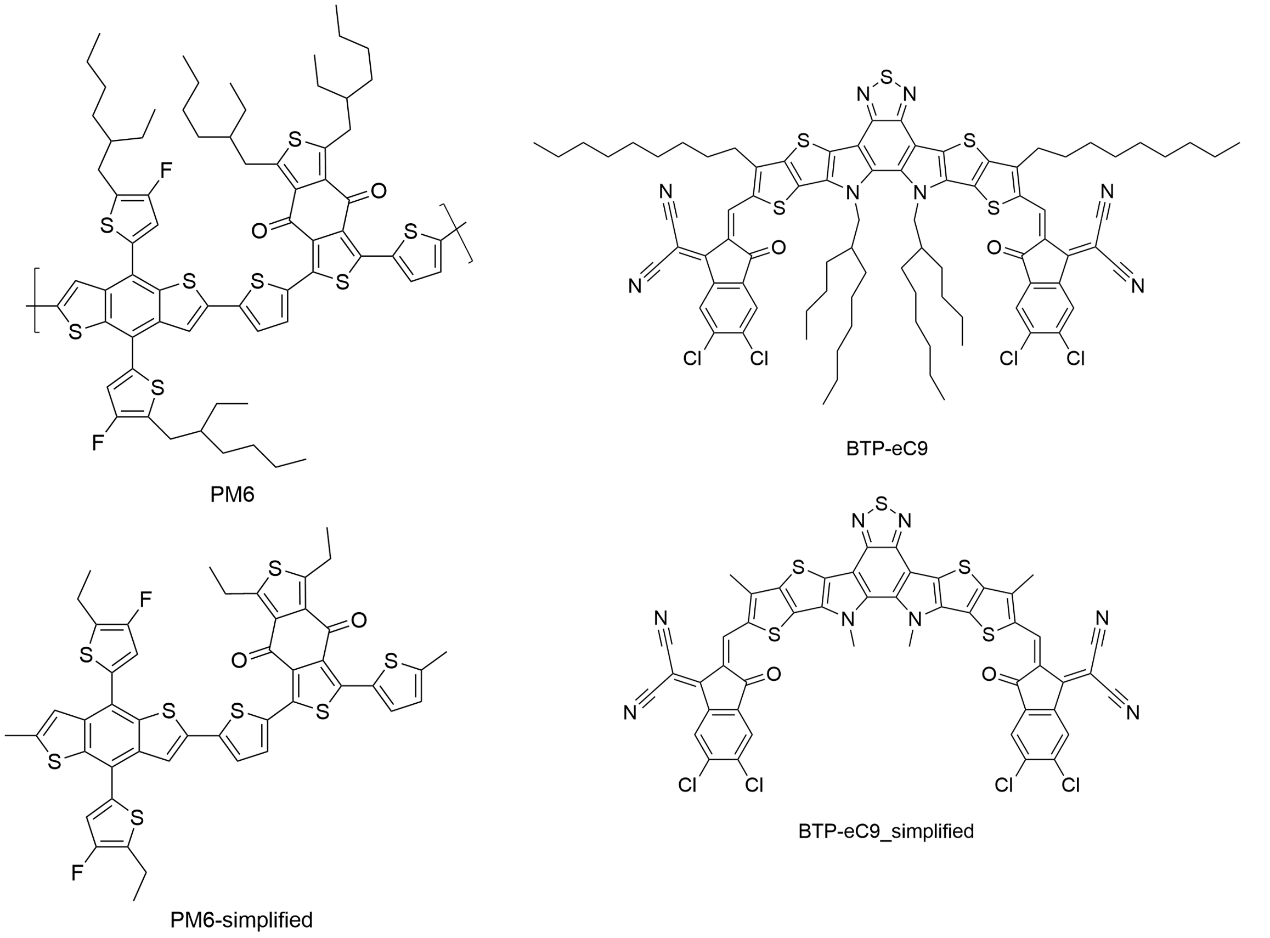


Figure S9. The molecular formula of the donor (PM6) and acceptor (BTP-eC9) and the simplified structure for convenience of theoretical calculation.^10, 11^


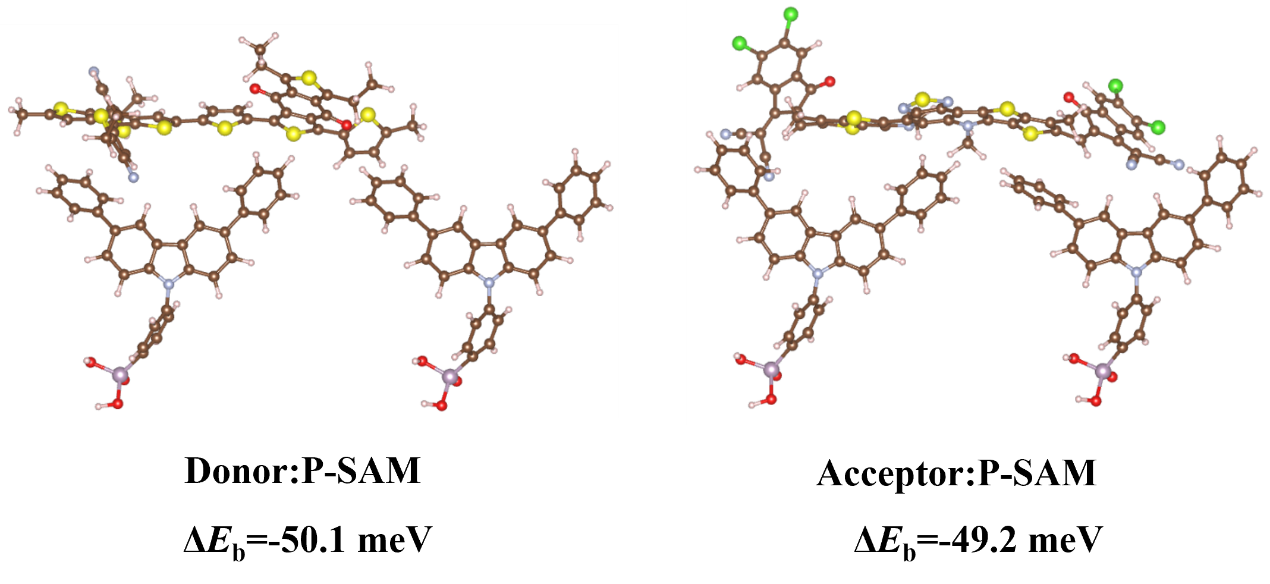


Figure S10. DFT calculated binding energies between simplified PM6 donor/ BTP-eC9 acceptor molecules and P-SAM.


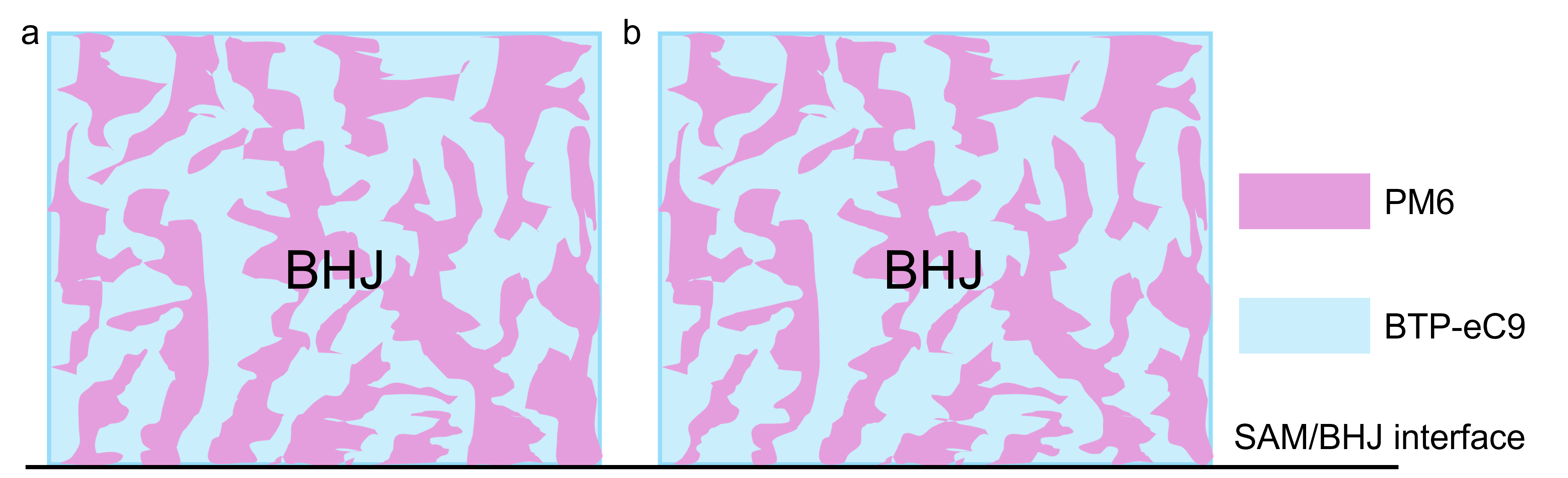


Figure S11. Schematic diagram of BHJ morphology near the buried interface, where (a) corresponds to a higher F/Cl element ratio (such as N-SAM modified structure) and (b) corresponds to a lower F/Cl element ratio (such as C-SAM modified structure).


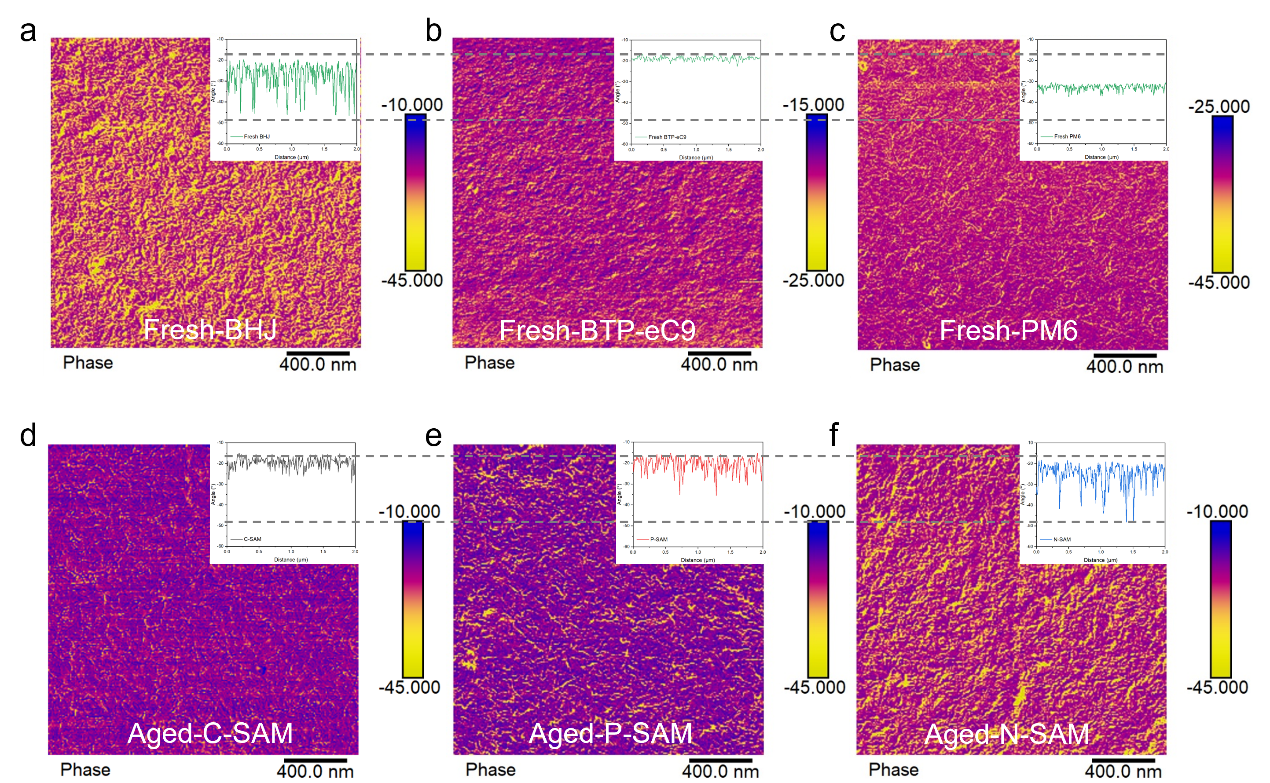


Figure S12. AFM phase image for the buried interface of BHJ/pure donor or acceptor. a) Fresh BHJ on ITO. b) Fresh BTP-eC9 on ITO. c) Fresh PM6 on ITO. d) aged BHJ on C-SAM modified ITO. e) Aged BHJ on P-SAM modified ITO. f) Aged BHJ on N-SAM modified ITO. The upper right corner is the corresponding line extraction result along the width direction of the phase images (with a range of -10°~-60°).


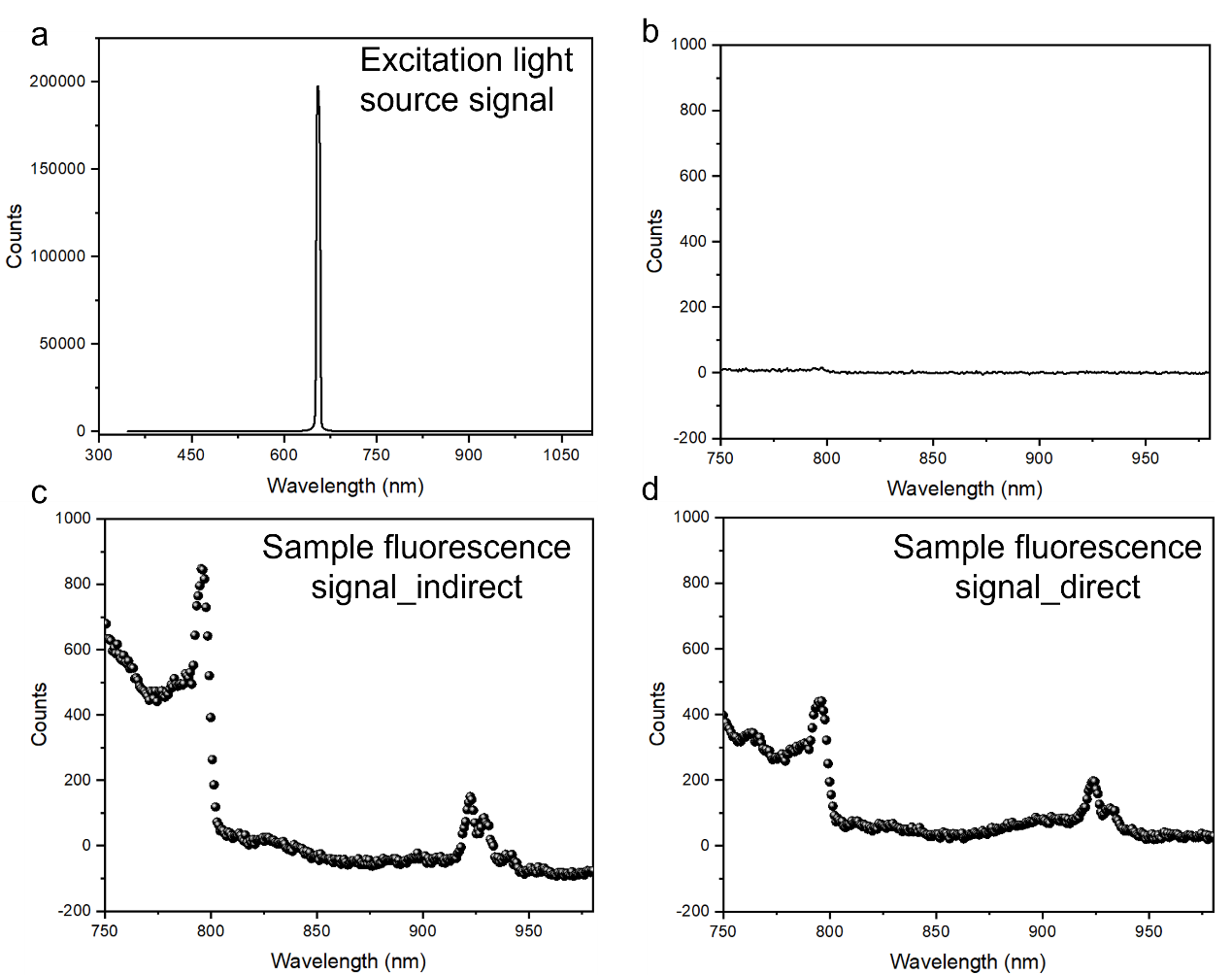


Figure S13. Spectra for PLQY calculations.^[2]^ a) Spectra for the Laser signal. b) Fluorescence signal without any samples. c) Fluorescence signal from ITO/SAM/BHJ samples with the Laser incident indirectly. d) Fluorescence signal from ITO/SAM/BHJ samples with the Laser incident directly. The photoluminescence peak around 790 nm is from the donor, and the photoluminescence peak around 925 nm is from the acceptor.


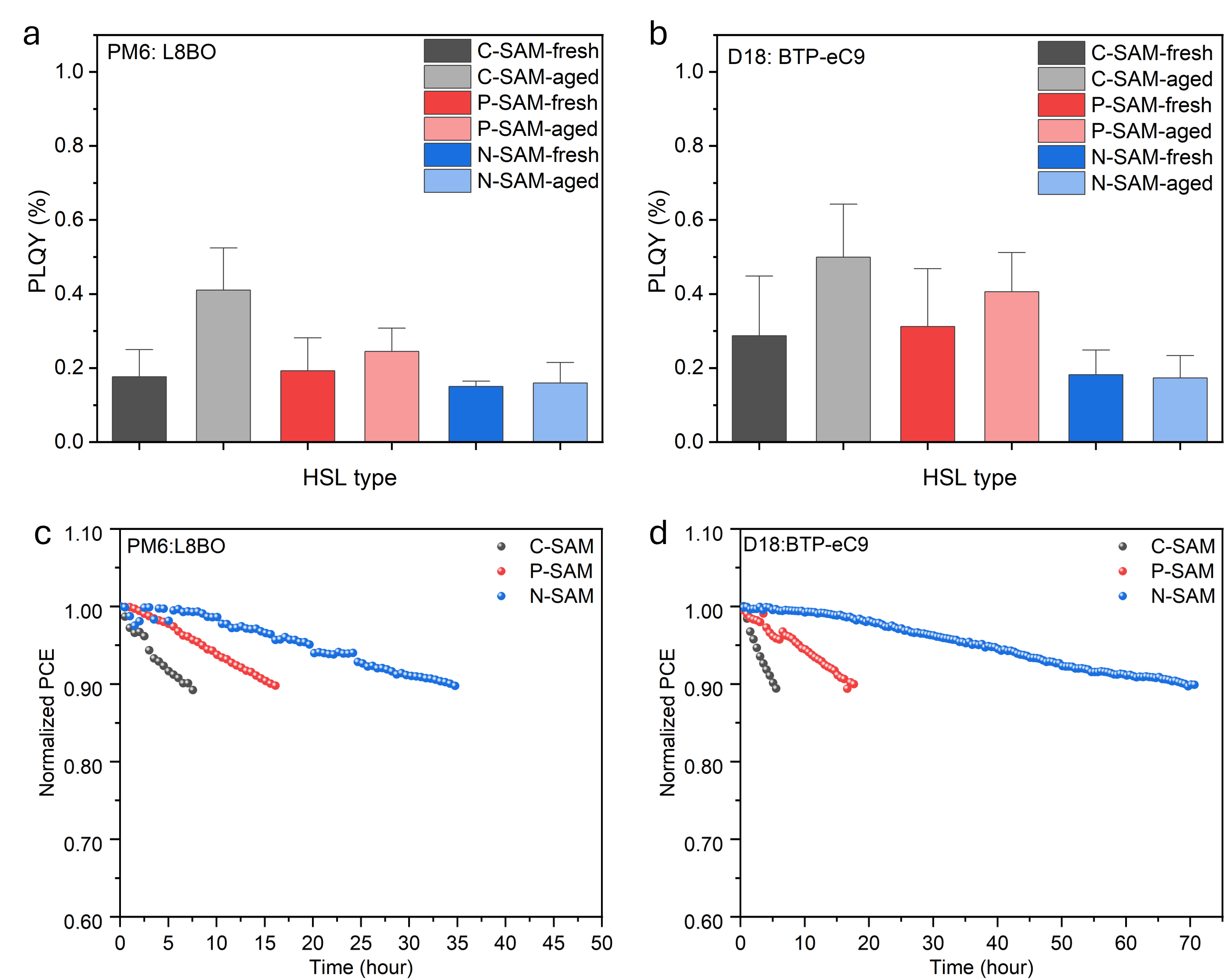


Figure S14. PLQY of fresh and thermally aged ITO/SAM/BHJ structures for PM6:L8BO BHJ system (a) and D18:BTP-eC9 system (b). MPP tracking results for devices based on PM6:L8BO BHJ system (c) and D18:BTP-eC9 system (d).

Figure S15. a) 2D TA profile of fresh and aged blend films on three SAM-modified substrates, showing spectral and temporal evolution of excited kinetics with key features labeled: GSB_A_: 850 nm; and GSB_D_: 630 nm. Dynamics of (b) GSB_A_, and (c) GSB_D_ in fresh and aged blend films on different SAM-modified substrates. All the data in are normalized to the minima of GSB_A_ at 0.1 ps, respectively. We used a pump pulse at 800 nm to selectively excite the acceptor BTP-eC9, resulting in the appearance of ground-state bleaching signals corresponding to BTP-eC9 (GSB_A_) at 850 nm. The hole transfer (HT) process was characterized by the decrease of GSB_A_, accompanied by the increase of ground state bleaching of PM6 (GSB_D_; 630 nm). To evaluate the influence of different SAMs on the HT process, the TA kinetics were normalized to the minimum of GSB_A_ for each sample, and the rise dynamics of GSB_D_ were compared. As illustrated in Figure 1b, c, the GSB_A_ show similar decay behaviors across all samples, whereas the rise dynamics of GSB_D_ exhibit the order of HT efficiency as N-SAM > P-SAM > C-SAM, indicating that N-SAM facilitates faster and more efficient hole transfer from BTP-eC9 to PM6, thereby mitigating geminate recombination. Notably, even after aging (Figure 1c), the same trend is preserved among the three SAMs, suggesting that the SAM-induced interfacial properties remain effective in promoting charge extraction over time.


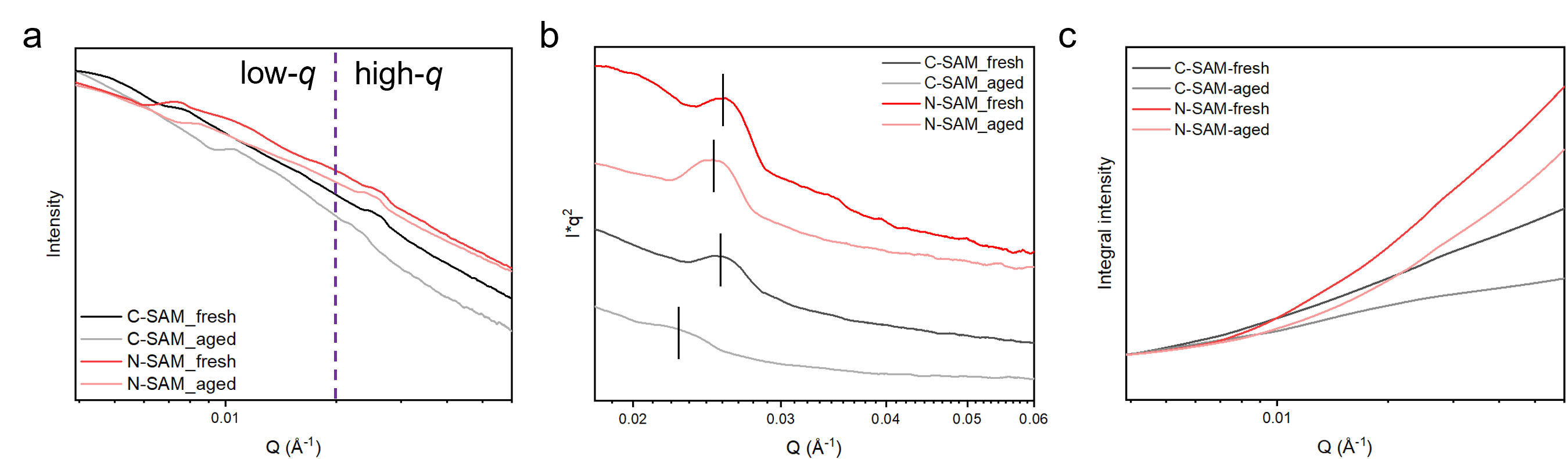


Figure S16. a) RSoXS profiles of fresh and aged BHJ films on C-SAM and N-SAM-modified substrates. b) Intensity·q² plots derived from the RSoXS date for domain size analysis. c) Integrated RSoXS intensity used to assess domain purity.


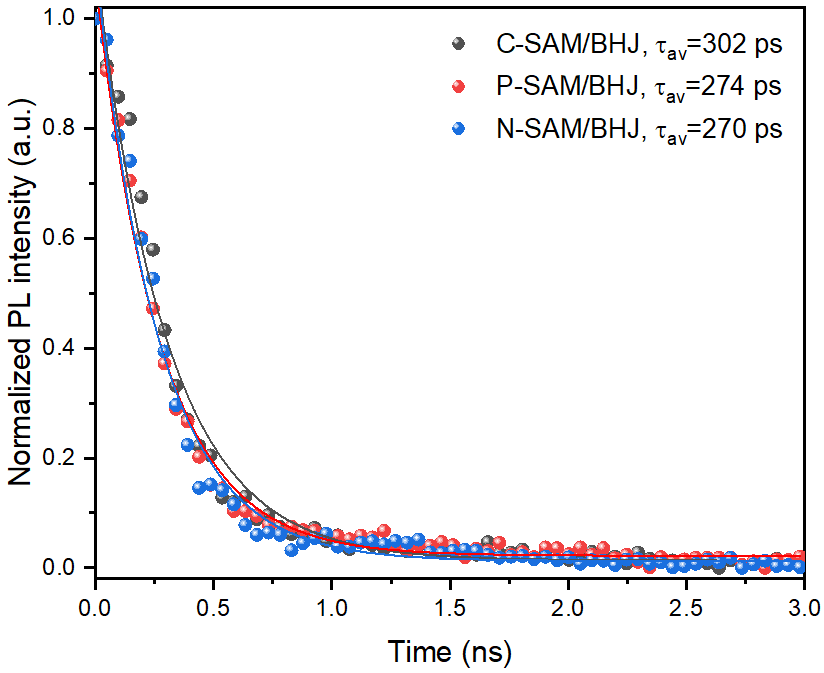


Figure S17. Time-resolved photoluminescence spectroscopy (TRPL) results for charge carrier lifetime extraction. The lifetimes with C-SAM, P-SAM, and N-SAM as hole-selective layer (HSL) samples are 302 ps, 274 ps, and 270 ps, respectively, which indicates a more efficient hole extraction with N-SAM as HSL.


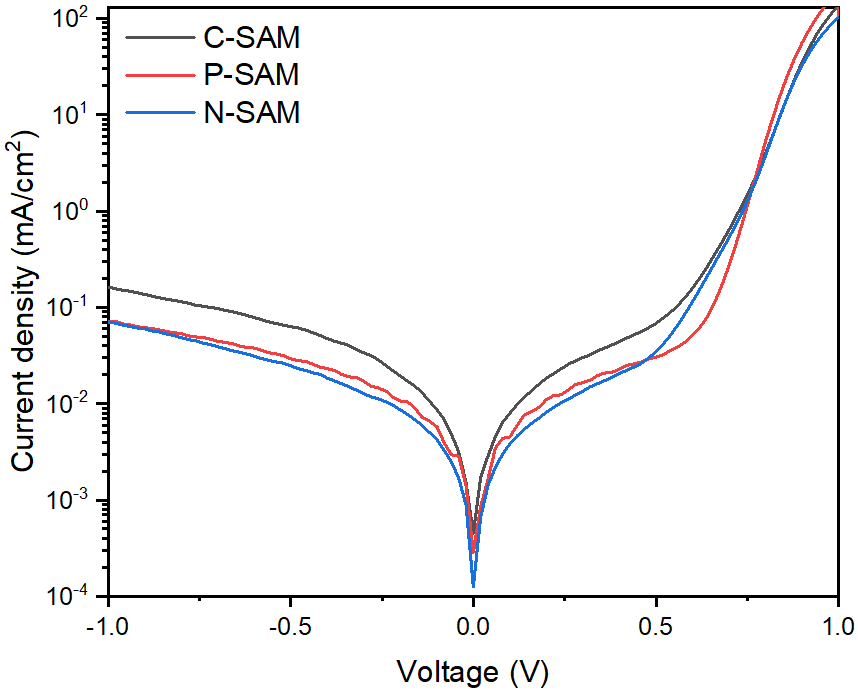


Figure S18. Dark current curves of organic solar cells with different SAMs as HSL.


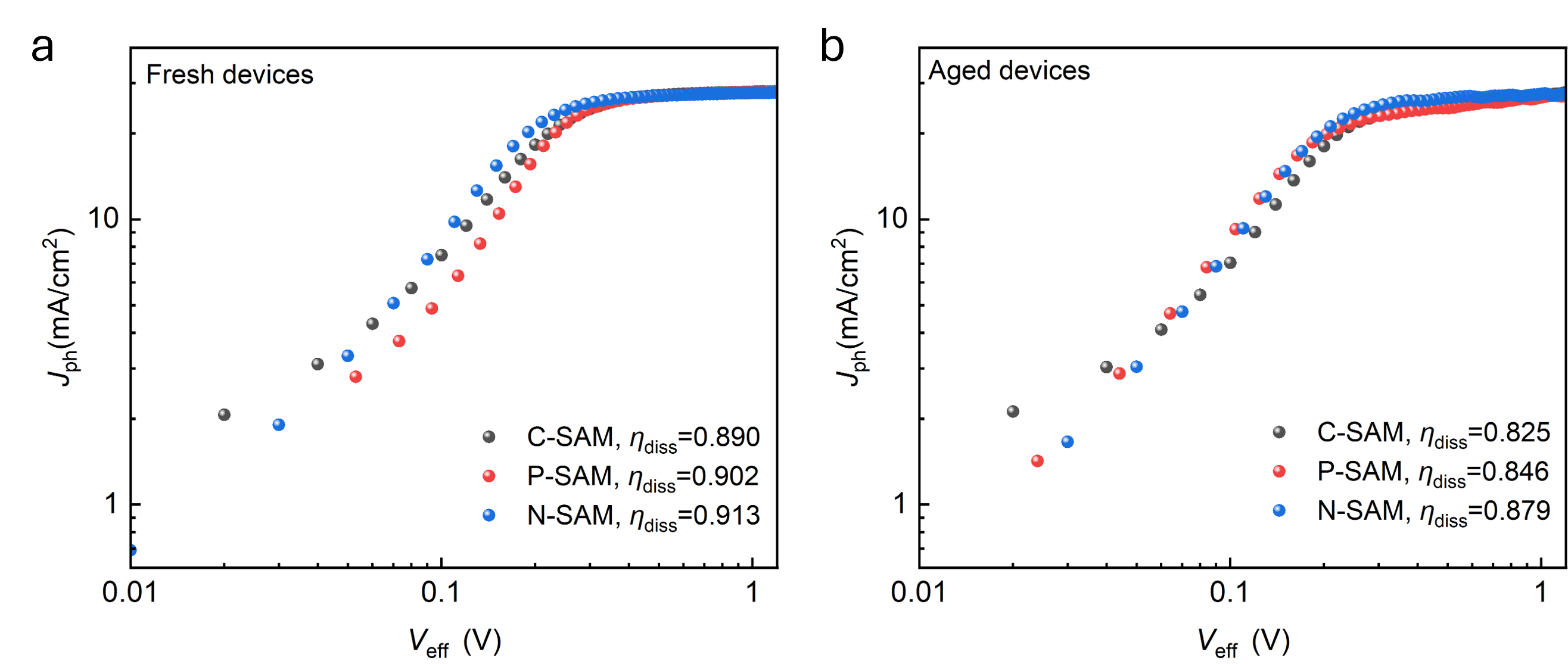


Figure S19. Photocurrent density (*J*_ph_) as a function of the effective voltage (*V*_eff_) for fresh devices (a) and aged devices (b). *J*_ph_ is defined as *J*_ph_=*J*_L_−*J*_D_, where *J*_L_ and *J*_D_ are the current densities in light and dark conditions, respectively. *V*_eff_ is calculated by *V*_eff_=*V*_0_−*V*_a_, *V*_0_ is the value of the voltage when *V*_ph_ is equal to 0, and *V*_a_ is the value of applied voltage. The exciton dissociation efficiency (*η*_diss_) is defined as *η*_diss_ =*J_SC_* /*J*_sat_, and *J*_sat_ represents the magnitude of the saturation current density.^12^


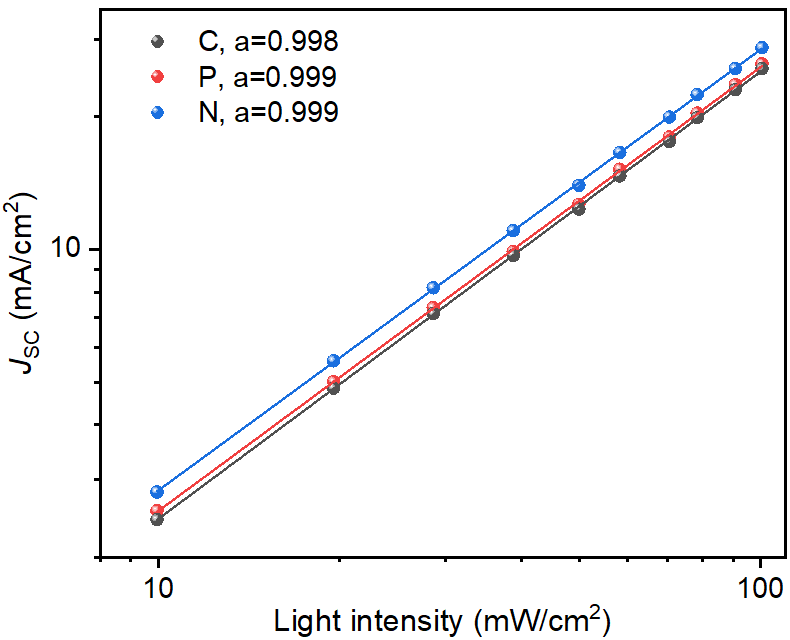


Figure S20. Dependence of short-circuit current density on light intensity of organic solar cells with different SAMs as HSL.


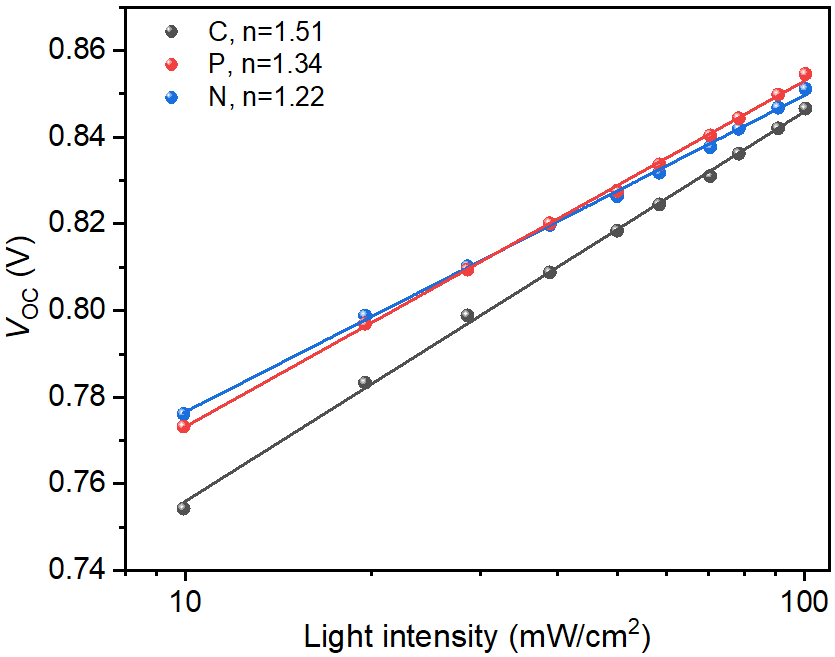


Figure S21. Dependence of open-circuit voltage on light intensity of organic solar cells with different SAMs as HSL.


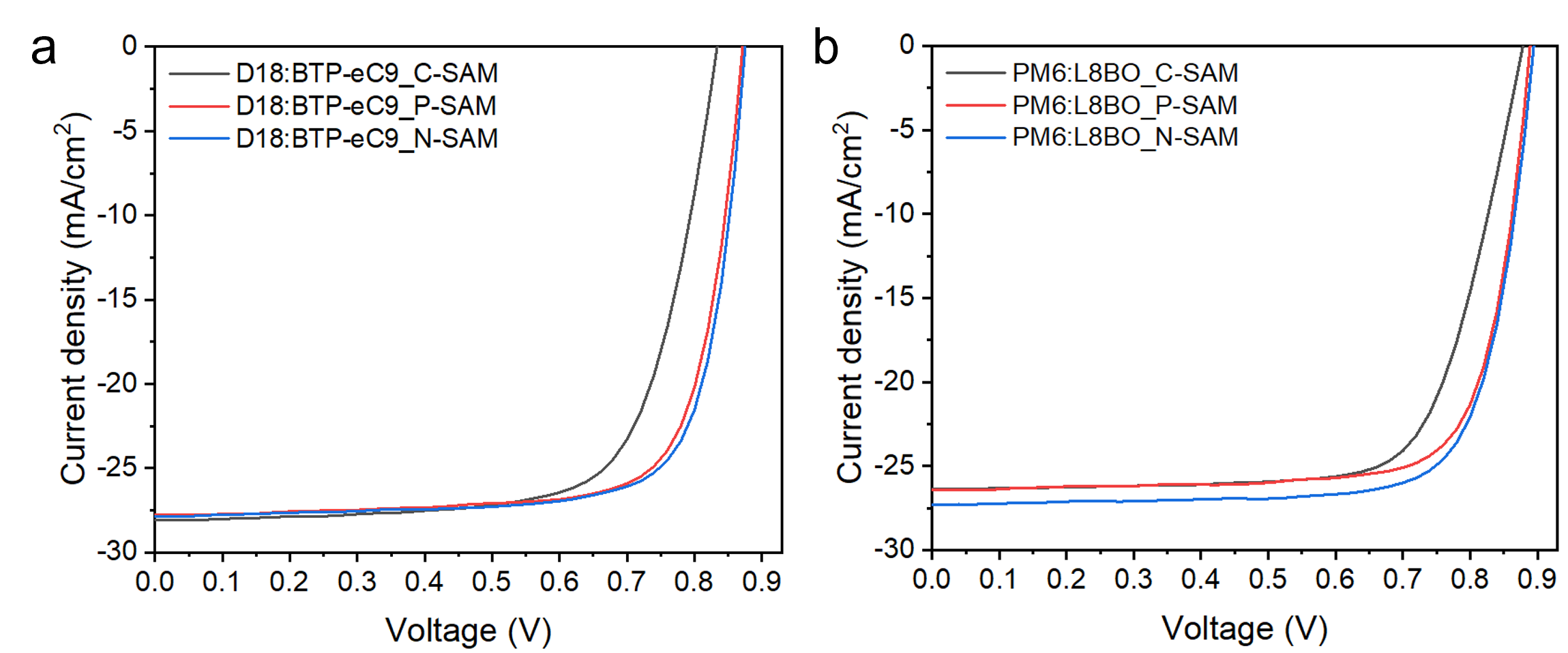


Figure S22. *J-V* curves of devices with different SAMs as HSL based on D18:BTP-eC9 BHJ (a) and PM6:L8BO BHJ (b).


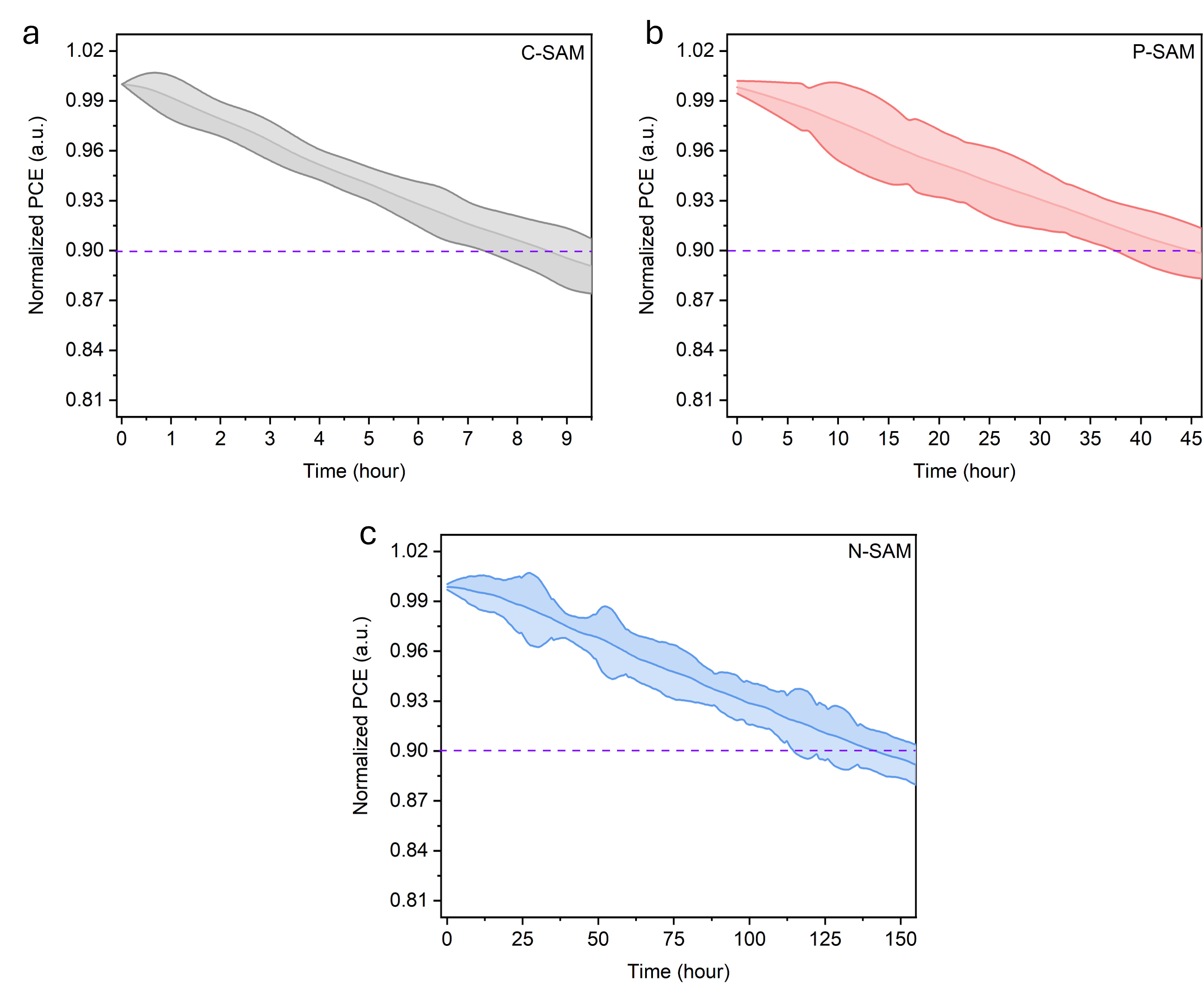


Figure S23. MPP tracking measurements for C-SAM (a), P-SAM (b) and N-SAM (c) based devices, respectively.^13, 14^ The average lifetimes with standard deviations are as follows: 141.7 ± 7.4 h (N-SAM), 44.9 ± 2.8 h (P-SAM), and 8.68 ± 0.61 h (N-SAM).


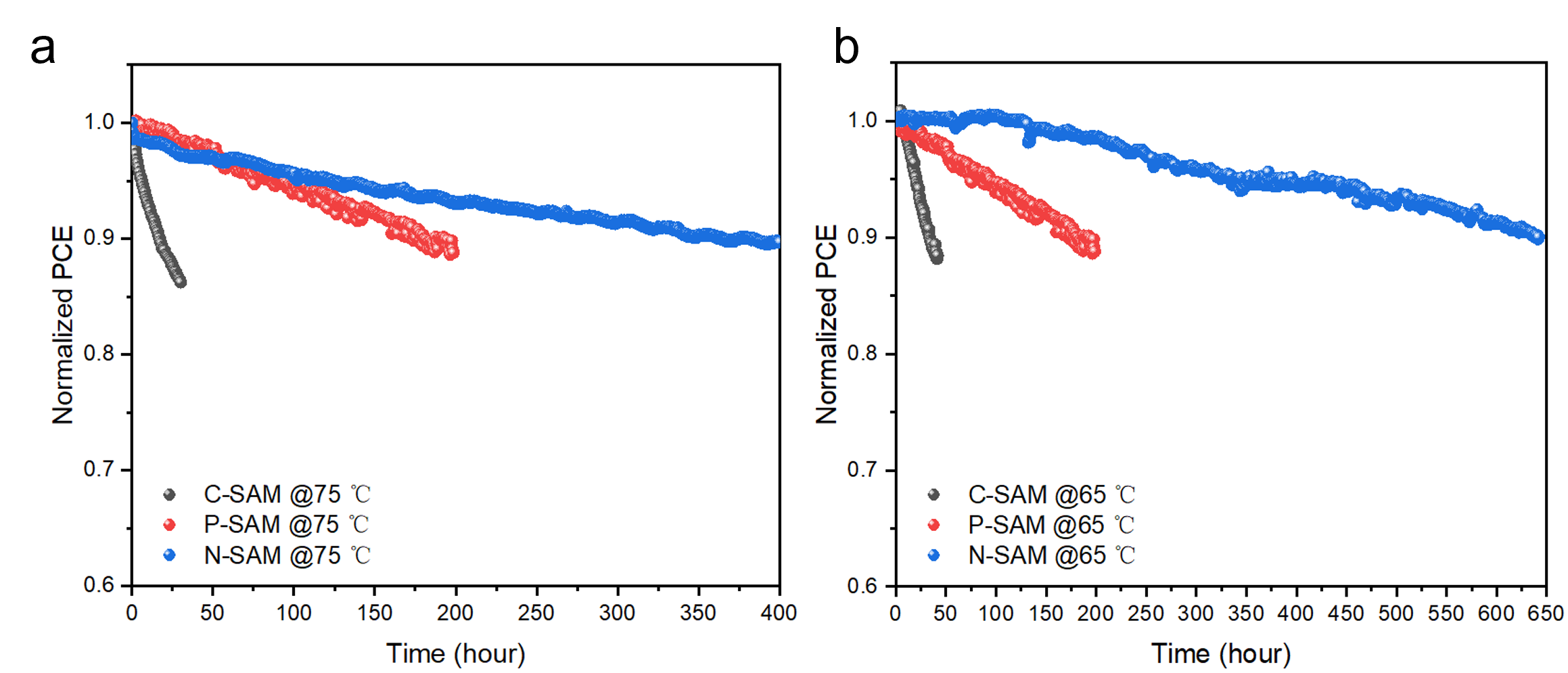


Figure S24. PCE decay in maximum power point tracking at different temperatures for devices with different SAMs HSL. a) 75 °C, b) 65 °C.

**Tables**

Table S1. Photovoltaic parameters of OPV devices under the illumination of AM 1.5G, 100mW cm^−2^.

| Solvents | *V*_OC_ (V) | FF (%) | *J*_SC_ (mA/cm^2^) | PCE（%） |
| --- | --- | --- | --- | --- |
| THF | 0.856 | 68.49 | 23.56 | 13.86 |
| MeOH&THF | 0.857 | 77.52 | 28.76 | 19.11 |
| EtOH&THF | 0.864 | 77.88 | 29.33 | 19.73 |

Table S2. Atomic concentration determined by XPS for SAMs modified ITO substrate.

| Atomic ratio (%) | C | In | N | O | P | P/In |
| --- | --- | --- | --- | --- | --- | --- |
| C-SAM | 34.96 | 24.6 | 2.04 | 36.79 | 1.61 | 6.54% |
| P-SAM | 53.63 | 16.17 | 2.27 | 26.36 | 1.56 | 9.65% |
| N-SAM | 55.14 | 15.79 | 1.47 | 25.94 | 1.66 | 10.51% |

Table S3. Domain parameters of BHJ morphology extracted from RSoXS measurements.

|  | Domain size (nm) | Relative domain purity |
| --- | --- | --- |
| C-SAM_fresh | 24.7 | 73.9% |
| C-SAM_aged | 27.8 | 53.5% |
| N-SAM_fresh | 24.6 | 100% |
| N-SAM_aged | 25.2 | 87.7% |

Table S4. Photovoltaic parameters of OPV devices under the illumination of AM 1.5G, 100mW cm^−2^.

| SAM | BHJ | *V*_OC_ (V) | FF (%) | *J*_SC_ (mA/cm^2^) | PCE (%) |
| --- | --- | --- | --- | --- | --- |
| C-SAM | D18:BTP-eC9 | 0.834  (0.829±0.008) | 71.18  (70.8±0.47) | 28.09  (27.65±0.52) | 16.67^a)^  (16.22±0.20)^b)^ |
| P-SAM | D18:BTP-eC9 | 0.871  (0.867±0.003) | 76.11  (75.57±0.37) | 27.78  (27.62±0.30) | 18.42  (18.09±0.25) |
| N-SAM | D18:BTP-eC9 | 0.875  (0.873±0.002) | 76.65  (75.85±0.80) | 27.88  (27.74±0.28) | 18.71  (18.36±0.20) |
| C-SAM | PM6:L8BO | 0.878  (0.864±0.013) | 72.78  (71.60±0.95) | 26.38  (26.42±0.41) | 16.86  (16.35±0.23) |
| P-SAM | PM6:L8BO | 0.889  (0.885±0.005) | 76.93  (75.79±1.03) | 26.40  (26.32±0.51) | 18.06  (17.65±0.22) |
| N-SAM | PM6:L8BO | 0.895  (0.897±0.006) | 76.59  (77.53±1.04) | 27.28  (26.30±0.46) | 18.69  (18.28±0.29) |

^a))^ The maximal values; ^b))^ average values with standard deviation derived from 10 independent devices.

**Reference**

(1) Zhang, N.; Jiang, W.; An, Y.; Liu, Q.; Du, G.; Xia, T.; Chen, D.; Wong, C. T.; Zeng, X. C.; Lin, F. R.; et al. Enhancing UV Stability and Charge Extraction in Organic Solar Cells with Phenyl‐Linked Aromatic Self‐Assembled Monolayer. *Advanced Functional Materials* **2025**. DOI: 10.1002/adfm.202423178.

(2) Gu, J.; You, X.; Tao, C.; Li, J.; Gerbaud, V. Energy-Saving Reduced-Pressure Extractive Distillation with Heat Integration for Separating the Biazeotropic Ternary Mixture Tetrahydrofuran–Methanol–Water. *Industrial & Engineering Chemistry Research* **2018**, *57* (40), 13498-13510. DOI: 10.1021/acs.iecr.8b03123.

(3) Kong, Z. Y.; Yang, A.; Saptoro, A.; Sunarso, J. Revisiting the binary azeotropic separation containing tetrahydrofuran and ethanol: Design and control of extractive distillation using dimethyl sulfoxide as alternative solvent. *Digital Chemical Engineering* **2022**, *5*. DOI: 10.1016/j.dche.2022.100060.

(4) de Mello, J. C.; Wittmann, H. F.; Friend, R. H. An improved experimental determination of external photoluminescence quantum efficiency. *Adv. Mater.* **2004**, *9* (3), 230-232. DOI: 10.1002/adma.19970090308.

(5) Kühne, T. D.; Iannuzzi, M.; Del Ben, M.; Rybkin, V. V.; Seewald, P.; Stein, F.; Laino, T.; Khaliullin, R. Z.; Schütt, O.; Schiffmann, F.; et al. CP2K: An electronic structure and molecular dynamics software package - Quickstep: Efficient and accurate electronic structure calculations. *J. Chem. Phys.* **2020**, *152* (19), 194103. DOI: 10.1063/5.0007045.

(6) van Setten, M. J.; Giantomassi, M.; Bousquet, E.; Verstraete, M. J.; Hamann, D. R.; Gonze, X.; Rignanese, G. M. The PseudoDojo: Training and grading a 85 element optimized norm-conserving pseudopotential table. *Comput. Phys. Commun.* **2018**, *226*, 39-54. DOI: <https://doi.org/10.1016/j.cpc.2018.01.012>.

(7) Humphrey, W.; Dalke, A.; Schulten, K. VMD: Visual molecular dynamics. *Journal of Molecular Graphics* **1996**, *14* (1), 33-38. DOI: https://doi.org/10.1016/0263-7855(96)00018-5.

(8) Lu, T.; Chen, F. Multiwfn: A multifunctional wavefunction analyzer. *J. Comput. Chem.* **2012**, *33* (5), 580-592. DOI: <https://doi.org/10.1002/jcc.22885>.

(9) Lu, T. A comprehensive electron wavefunction analysis toolbox for chemists, Multiwfn. *The Journal of Chemical Physics* **2024**, *161* (8). DOI: 10.1063/5.0216272.

(10) Huang, J.; Chen, T.; Mei, L.; Wang, M.; Zhu, Y.; Cui, J.; Ouyang, Y.; Pan, Y.; Bi, Z.; Ma, W.; et al. On the role of asymmetric molecular geometry in high-performance organic solar cells. *Nat Commun* **2024**, *15* (1), 3287. DOI: 10.1038/s41467-024-47707-5.

(11) Duan, T.; Feng, W.; Li, Y.; Li, Z.; Zhang, Z.; Liang, H.; Chen, H.; Zhong, C.; Jeong, S.; Yang, C. Electronic configuration tuning of centrally extended non‐fullerene acceptors enabling organic solar cells with efficiency approaching 19%. *Angewandte Chemie* **2023**, *135* (42), e202308832.

(12) Ma, R.; Tao, Y.; Chen, Y.; Liu, T.; Luo, Z.; Guo, Y.; Xiao, Y.; Fang, J.; Zhang, G.; Li, X.; et al. Achieving 16.68% efficiency ternary as-cast organic solar cells. *Science China Chemistry* **2021**, *64* (4), 581-589. DOI: 10.1007/s11426-020-9912-0.

(13) Reese, M. O.; Gevorgyan, S. A.; Jørgensen, M.; Bundgaard, E.; Kurtz, S. R.; Ginley, D. S.; Olson, D. C.; Lloyd, M. T.; Morvillo, P.; Katz, E. A.; et al. Consensus stability testing protocols for organic photovoltaic materials and devices. *Sol. Energy Mater. Sol. Cells* **2011**, *95* (5), 1253-1267. DOI: 10.1016/j.solmat.2011.01.036.

(14) Khenkin, M. V.; Katz, E. A.; Abate, A.; Bardizza, G.; Berry, J. J.; Brabec, C.; Brunetti, F.; Bulović, V.; Burlingame, Q.; Di Carlo, A.; et al. Consensus statement for stability assessment and reporting for perovskite photovoltaics based on ISOS procedures. *Nature Energy* **2020**, *5* (1), 35-49. DOI: 10.1038/s41560-019-0529-5.
